# BLU-451, a CNS-Active, Potent, and Selective Small-Molecule Inhibitor against Uncommon *EGFR* Mutations

**DOI:** 10.1158/1535-7163.MCT-25-1056

**Published:** 2026-05-13

**Authors:** Yves A. Millet, Alexander I. Spira, Christina S. Baik, Min Hee Hong, Elaine Shum, Jyoti D. Patel, Danny Nguyen, Pasi A. Jänne, Zofia Piotrowska, Natasha B. Leighl, Shingo Matsumoto, Lova Sun, Tsung-Ying Yang, Tatsuya Yoshida, Monique B. Nilsson, John V. Heymach, Brion W. Murray, Jeffrey Keats, Stephanie Lee, John Hsieh, Alena Zalutskaya, Xiuning Le

**Affiliations:** 1 Blueprint Medicines Corporation, Cambridge, Massachusetts.; 2 Virginia Cancer Specialists Research Institute, Fairfax, Virginia.; 3 Division of Hematology and Oncology, University of Washington School of Medicine, Seattle, Washington.; 4 Yonsei Cancer Center, Severance Hospital, Yonsei University College of Medicine, Seoul, Republic of Korea.; 5 NYU Perlmutter Cancer Center, NYU Grossman School of Medicine, New York, New York.; 6 Tempus AI, Chicago, Illinois.; 7 Department of Medical Oncology and Therapeutics Research, City of Hope, Irvine, California.; 8 Dana-Farber Cancer Institute, Boston, Massachusetts.; 9 Massachusetts General Hospital Cancer Center, Boston, Massachusetts.; 10 Division of Medical Oncology, Princess Margaret Cancer Centre, Toronto, Canada.; 11 Department of Thoracic Oncology, National Cancer Center Hospital East, Kashiwa, Japan.; 12 Department of Medicine, Hospital of the University of Pennsylvania, Philadelphia, Pennsylvania.; 13 Department of Chest Medicine, Taichung Veterans General Hospital, Taichung, Taiwan.; 14 Department of Thoracic Oncology, National Cancer Center Hospital, Tokyo, Japan.; 15 Department of Thoracic-Head and Neck Medical Oncology, The University of Texas MD Anderson Cancer Center, Houston, Texas.

## Abstract

BLU-451 is a potent and selective tyrosine kinase inhibitor designed to target uncommon epidermal growth factor receptor (*EGFR*) mutations, spare wild-type (WT) EGFR, and be active in the central nervous system (CNS). *In vitro* EGFR enzyme assays and engineered cell line models revealed selectivity and antiproliferative potency of BLU-451 against a wide range of *EGFR* mutations in non–small cell lung cancer, including common mutations, atypical mutations, and exon 20 insertions. Particularly, BLU-451 demonstrated robust antitumor activity in *EGFR* exon 20 insertion cell-derived and patient-derived xenograft models and was well tolerated in animal studies. Pharmacokinetic and pharmacodynamic analyses showed that BLU-451 suppressed exon 20 insertion phosphorylated *EGFR* expression levels within tumor tissues but not in skin and intestinal tissues, potentially indicative of lower toxicity associated with WT EGFR. In intracranial tumor models with imaging-based analyses, BLU-451 showed CNS antitumor efficacy. Early clinical data from patients enrolled in the phase I portion of the phase I/II CONCERTO trial (NCT05241873) demonstrated the overall clinical potential of BLU-451, including its CNS antitumor activity in patients with *EGFR* exon 20 insertions and atypical mutations. Initial data suggest that BLU-451 is an active molecule. Further evaluation of the safety profile and pharmacokinetic properties of BLU-451 is needed.

## Introduction

In non–small cell lung cancer (NSCLC), uncommon epidermal growth factor (EGF) receptor (*EGFR*) mutations, including exon 20 insertions and atypical variants such as G719X, L861Q, and S768I, present unique challenges and opportunities for targeted therapy (1). Uncommon *EGFR* mutations are highly heterogeneous and can be classified based on the structure and function as classical-like, T790M-like, and P-loop and α-helix compressing mutations (PACC); and exon 20 subgroups, which may aid in predicting the drug sensitivity and patient outcomes (2, 3). *EGFR* exon 20 insertion mutations include more than 80 unique variants, including D770insX, V769insX, A763insFQEA, H773insNPH, and V774insHV, which are characterized by in-frame insertions of one to seven amino acids (represented as X; e.g., D770insX), pushing the EGFR C helix into a constitutively active kinase conformation (1, 4). The resulting steric hindrance to the EGFR drug–binding site leads to ineffective binding of most approved EGFR tyrosine kinase inhibitors (TKI; refs. 1, 4). Therefore, patients with these mutations typically exhibit lower response and survival rates with current EGFR-TKIs (4, 5). Furthermore, central nervous system (CNS)–active TKIs of uncommon *EGFR* mutations are needed, given that approximately 25% of patients with uncommon *EGFR* mutations have CNS metastases, and a substantial proportion of patients develop CNS disease at the time of progression (6–9).

Currently, there are limited treatment options for patients with metastatic NSCLC harboring uncommon *EGFR* mutations (5). Until recently, platinum-based chemotherapy was the first-line standard of care for *EGFR* exon 20 insertions (4). Amivantamab, a humanized anti-EGFR/c-MET bispecific antibody, was conditionally approved in the United States for patients with relapsed exon 20 insertion *EGFR*-mutant NSCLC (4, 10). After progression on chemotherapy, amivantamab was observed to have an overall response rate of 40% and median duration of response of 11.1 months, but no data on CNS overall response rate were available because patients with active brain metastases were excluded from the trial (10, 11). In 2024, the Food and Drug Administration (FDA) approved the use of amivantamab in combination with chemotherapy as standard of care for first-line treatment in patients with *EGFR* exon 20 insertions based on data from the phase III PAPILLON trial that reported a progression-free survival of 11.4 months for the combination cohort compared with 6.7 months for patients on chemotherapy alone (12, 13). However, both amivantamab monotherapy and amivantamab in combination with chemotherapy are associated with a high frequency of toxicities (4). In the PAPILLON trial, grade ≥3 adverse events (AEs) were observed in 75% of patients who received amivantamab in combination with chemotherapy, with 69% of AEs leading to dose interruptions, 48% of AEs leading to dose reductions, and 24% of AEs leading to dose discontinuations (14). Recently, the FDA granted accelerated approval to the first TKI, sunvozertinib, for adult patients with locally advanced or metastatic NSCLC with *EGFR* exon 20 insertion mutations in which the disease has progressed on or after platinum-based chemotherapy, based on the results from the WU-KONG1B trial (15).

For patients with certain atypical “nonresistant” *EGFR* mutations, the only currently approved treatment option is the second-generation TKI afatinib, which has an overall response rate of 71.1% and a median overall survival of 19.4 months in a select group of atypical mutations (16). However, afatinib is associated with severe AEs leading to high rates of dose reduction and discontinuation, limiting its clinical utility (16), and instead, the third-generation EGFR inhibitor osimertinib is frequently used in the treatment of atypical *EGFR* mutations because of favorable outcomes in some cohorts and its CNS penetration. However, clinical data about osimertinib efficacy remain inconclusive, and osimertinib does not have regulatory approval for use in atypical *EGFR*-mutated NSCLC (4, 16, 17). There is, therefore, a need for EGFR-TKIs that specifically target both exon 20 insertion and atypical mutations with fewer treatment-limiting AEs and meaningful CNS activity (1, 4, 16).

BLU-451 is a CNS-active, wild-type (WT) EGFR-sparing, covalent TKI targeting uncommon *EGFR* mutations (18, 19). In this study, we present preclinical data to demonstrate the potency and selectivity of BLU-451 against EGFR with exon 20 insertion and atypical mutations. Using cell-derived xenograft and patient-derived xenograft (PDX) models as well as intracranial tumor models, we demonstrate the *in vivo* antitumor efficacy and CNS activity of BLU-451. Initial case reports from patients enrolled in the phase I portion of the phase I/II CONCERTO trial (NCT05241873, now discontinued) demonstrate the clinical potential of BLU-451.

## Materials and Methods

### Synthesis of BLU-451

BLU-451 was synthesized as described in the Patent Publication WO2023/215431 (20).

### Biochemical characterization of BLU-451 inhibition of WT and mutant EGFR

The inhibitory effects of BLU-451 and osimertinib were determined by measuring the enzymatic activity of the WT EGFR and mutants to phosphorylate fluorescent substrate (5-FAM-EEPLYWSFPAKKK-CONH_2_, ProfilerPro Kinase Peptide Substrate 22, PerkinElmer, 2.5 μmol/L) in the presence of 1 mmol/L adenosine triphosphate (ATP) at varying concentrations of the testing compound. The EGFR enzyme reaction buffer contains 10 mmol/L MgCl_2_, 0.015% Brij-35, 1 mmol/L dithiothreitol, 1% dimethyl sulfoxide (DMSO), and 100 mmol/L 4-(2-hydroxyethyl)-1-piperazineethanesulfonic acid (HEPES), pH 7.5, at 25°C. The kinase reaction was activated by mixing the enzyme–ATP solution, the peptide substrate, and BLU-451. The reactions proceeded for 60 minutes and were terminated with 35 mmol/L ethylenediaminetetraacetic acid (EDTA). The analysis of the proportion of phosphorylated substrate peptide was performed automatically on the Caliper EZ Reader 2 (PerkinElmer), in which the phosphorylated peptide (product) and substrate were electrophoretically separated and measured. The percent activity was plotted against log concentration of compound to generate an apparent half-maximal inhibitory concentration (IC_50_) using a four-parameter fit.

### Kinase profiling assays

BLU-451 was tested at 1 μmol/L in the KINOMEscan screening platform, which uses an active site-directed competitive binding assay to measure interactions with a panel of human kinases and disease-relevant mutant variants (DiscoverX Eurofins). The hits with the remaining kinase activity (% Ctrl) were transformed to % inhibition with the formula % inhibition = 100 − %Ctrl.

The enzymatic kinase profiling was performed at Reaction Biology Corporation following previously described procedures (21). Specific kinase/substrates with required cofactors were prepared in reaction buffer (20 mmol/L HEPES, pH 7.5, 10 mmol/L MgCl_2_, 1 mmol/L ethylene glycol tetraacetic acid, 0.02% Brij-35, 0.02 mg/mL bovine serum albumin, 0.1 mmol/L Na_3_VO_4_, 2 mmol/L dithiothreitol, and 1% DMSO). A concentration of 1 μmol/L BLU-451 was delivered into the reaction, followed 20 minutes later by the addition of a mixture of ATP (Sigma) and g-[^33^P]-ATP (PerkinElmer) to a final concentration of 10 μmol/L. The reactions were carried out at room temperature for 120 minutes, followed by spotting of the reactions onto P81 ion exchange filter paper (Whatman Inc.). Unbound phosphate was removed by extensive washing of filters in 0.75% phosphoric acid. After subtraction of background derived from control reactions containing inactive enzyme, kinase activity data were expressed as the percent remaining kinase activity in test samples compared with vehicle (DMSO) reactions.

### Cell lines

The A431 cells (American Type Culture Collection, RRID: CVCL_0037, biological sex: female) were grown in Dulbecco’s Modified Eagle Medium (DMEM) with 10% fetal bovine serum (FBS) and 1× penicillin–streptomycin (P/S) media. The PC9 cells (European Collection of Authenticated Cell Cultures, RRID: CVCL_B260, biological sex: male) were grown in RPMI 1640 medium with 10% FBS and 1× P/S media. The engineered Ba/F3 cells (RRID: CVCL_0161, biological sex: not specified) expressing D770insNPG, D770insSVD, H773insNPH, and V769insASV *EGFR* exon 20 insertions (used in the EGFR phosphorylation assays) were generated as previously described (22) and grown in RPMI 1640 medium with 10% FBS and 1× P/S. The G719A, G719S, and L861Q Ba/F3 cell lines (Kyinno Biotechnology) used in the EGFR phosphorylation assays were grown in RPMI 1640 with 10% FBS. The engineered Ba/F3 cells used in the cellular proliferation assays were generated and maintained as previously described (2). The A769insASV Ba/F3 cell line (Signosis) used in the mouse tumor model was grown in RPMI 1640 with 10% FBS and 1× P/S medium. The PC9-luciferase (PC9-Luc) cell lines used for the intracranial and intracarotid mouse tumor models were generated by stable transfection with the firefly luciferase gene and grown in RPMI 1640 with 10% FBS. The PC9-Luc cell line used in the intracarotid injection model was generated at ChemPartner.

### *In vitro* EGFR phosphorylation assays

BLU-451, osimertinib, and afatinib inhibition of EGFR phosphorylation in A431 and engineered Ba/F3 cell lines was assessed as previously described (22). Briefly, the cells were resuspended in fresh DMEM (with 10% FBS for PC9 and Ba/F3 cells) and seeded in the 384-well plates in technical duplicates. After 16 hours, the cells were treated with increasing doses of BLU-451, osimertinib, afatinib, or DMSO for 4 hours, and the inhibition of EGFR phosphorylation was assessed using the phospho-EGFR (Tyr1068) AlphaLISA SureFire Ultra kit (Revvity). For A431, the cells were stimulated with 30 ng/mL of EGF for 10 minutes after the 4-hour treatment period. The IC_50_ values were determined using XLfit and GraphPad Prism software. All the values are the geometric mean of at least two independent experiments. The WT-to-mutant ratios were calculated by dividing the geometric mean IC_50_ value of the A431 cell line expressing WT EGFR by the geometric mean IC_50_ values of mutant cell lines.

### *In vitro* cell proliferation assays

Cell proliferation assays with engineered Ba/F3 cells and IC_50_ calculations were performed as previously described (2). Briefly, the cells were resuspended in fresh RPMI medium with 10% FBS and seeded in 384-well plates in technical replicates. The Ba/F3 cells expressing the WT EGFR were supplemented with 10 ng/mL EGF. After 16 hours, the cells were treated with increasing doses of BLU-451, osimertinib, afatinib, or DMSO for 72 hours, and cell viability was assessed using CellTiter-Glo (Promega). The IC_50_ values were determined using GraphPad Prism software. The WT-to-mutant ratios were calculated by dividing the geometric mean IC_50_ values of mutant cell lines by the geometric mean IC_50_ value of the Ba/F3 cell line expressing WT EGFR.

### Animal care

All *in vivo* studies, including pharmacokinetic (PK)/pharmacodynamic (PD) evaluations and efficacy tumor models, were conducted in accordance with institutional guidelines. All animal studies were approved by the relevant Institutional Animal Care and Use Committee (IACUC), Institutional Animal Ethics Committee (IAEC), or equivalent ethics committee. Specifically, the IAEC at Jubilant Biosys Ltd. granted approval for the A769insASV Ba/F3 mouse PK/PD, brain-to-plasma ratio (in both mice and rats), and A769insASV Ba/F3 tumor model studies. The IACUCs at the following institutions granted approval for the following studies: Crown Bioscience (LU0387 PDX tumor model study); Charles River Laboratories (LXFE2478 PDX tumor model study); Blueprint Medicines (G719S Ba/F3 tumor model study); Pharmaron (intracranial PC9-Luc mouse tumor model study); and ChemPartner (intracarotid PC9-Luc mouse tumor model study). All procedures complied with applicable national and local regulations for the care and use of laboratory animals.

### A769insASV Ba/F3 mouse PK/PD study

The A769insASV Ba/F3 cells were grown in RPMI 1640 medium with 10% FBS and 1× P/S until they reached approximately 70% to 80% confluency. The cells were resuspended in 1:1 RPMI:Matrigel at 1.25 × 10^7^ cells/mL, and 200 μL (2.5 × 10^6^ cells) was injected into the right flank of female severe combined immunodeficient (SCID) beige mice (Vivo Bio Tech, RRID: IMSR_TAC:CBSCBG). The animals were randomized when the mean tumor size reached approximately 300 mm^3^. Six mice were randomized to the vehicle control (PEG-400: 20% Captisol, 20:80 v/v) and 12 to each BLU-451 group (10 and 50 mg/kg). A single dose was administered by oral gavage to the vehicle and treatment groups. Blood, tumor, large intestine, and skin samples were collected at 3 and 24 hours after dose for the vehicle group and at 1, 3, 8, and 24 hours for the BLU-451 groups (*n* = 3 per time point). Plasma was isolated and stored at −20°C. Tumors and skin tissue were cut into two different samples. The first sample was snap-frozen in liquid nitrogen for phosphorylated [phosphorylated EGFR (pEGFR)] analysis. The second sample was weighed, homogenized in 5× volume of phosphate-buffered saline (PBS), and stored at −20°C for BLU-451 quantification. For pEGFR analysis, the tissues were homogenized in 500 μL of tissue lysis buffer. The tissue lysates were then centrifuged at 14,000 rpm for 60 minutes at 4°C, and the supernatants were collected. The protein content in supernatants was estimated by bicinchoninic acid method, and 70 μg (tumor samples) or 120 μg (large intestine and skin samples) was loaded on an 8% SDS-PAGE gel for immunoblotting. pEGFR (Cell Signaling Technology, #2234) and β-actin (Cell Signaling Technology, #4970S) primary antibodies and the horseradish peroxidase (HRP)–linked antirabbit secondary antibodies (Cell Signaling Technology, #7074S) were used for Western blots. The blots were developed with Immobilon Forte Western HRP substrate in an Amersham ImageQuant 800 imaging system. The quantified levels of pEGFR and β-actin were analyzed using GraphPad Prism software. For BLU-451 bioanalysis, plasma, tumor, and skin homogenates were thawed on ice, and about 50 μL was added to 100% acetonitrile. The samples were then vortexed, and the supernatants were collected after centrifugation. A total of 5 or 10 μL of the supernatants was injected in liquid chromatography/tandem mass spectrometry (LC/MS-MS, API 4000) for analysis.

### Efflux transporter substrate activity assays

The substrate activity of BLU-451 for the human efflux transporters MDR1 and BCRP was evaluated in MDCKII-MDR1 (The Netherlands Cancer Institute, Netherlands) and MDCKII-BCRP (ReadyCell) cell monolayers. Briefly, transport of BLU-451 at 5 μmol/L across the cell monolayer in both the apical (A)-to-basal (B) and B-to-A directions was measured over 60 minutes at 37°C. The apparent permeability coefficients (P_app_ A to B and P_app_ B to A) were then determined, and the efflux ratio (P_app_ B to A/P_app_ A to B) was calculated.

### Brain-to-plasma ratio

The 7- to 8-week-old male BALB/c mice (Vivo Bio Tech, RRID: IMSR_TAC:BALB) were administered BLU-451 orally, either as a single 20 mg/kg dose or once daily for 5 days at 10 and 50 mg/kg. BLU-451 was formulated in DMSO:solutol:ethanol:saline pH 3 (10:5:5:80) and PEG-400: 20% Captisol (20:80) at 2 mg/mL in the single- and repeat-dose studies, respectively. The 7- to 8-week-old male Wistar rats (Vivo Bio Tech) were administered BLU-451 either orally as a single 30 mg/kg dose or by intravenous 5-hour continuous infusion at 20 mg/kg. BLU-451 was formulated in PEG-400: 20% Captisol (20:80) at 3 mg/mL. At collection times after dosing, the blood samples were drawn into disodium EDTA tubes; the animals were sacrificed, and the brains and cerebrospinal fluid (CSF; by cisterna magna, only for rats) samples were collected. Plasma and CSF samples were stored at −20°C. The brain samples were rinsed with PBS, blotted on tissue paper, weighed, homogenized in 5× volume of PBS, and stored at −20°C. On the day of the bioanalysis, plasma, brain, and CSF samples were thawed on ice, and about 50 μL was added to 400 μL of 100% acetonitrile. The samples were then vortexed, and the supernatants were collected after centrifugation. A total of 5 or 10 μL of the supernatants was injected in LC/MS-MS (API 4000) for analysis. When dosed orally, the area under the curve (AUC) values for BLU-451 brain, CSF, and plasma levels were used to calculate the K_p,brain_ (AUC_brain_/AUC_plasma_) and K_p,CSF_ (AUC_CSF_/AUC_plasma_) values. When dosed by intravenous continuous infusion, BLU-451 brain, CSF, and plasma levels at 5 hours were used to calculate K_p,brain_ and K_p,CSF_. The rat fraction unbound in plasma (f_u,plasma_) was determined by ultracentrifugation. The rat fraction unbound in brain (f_u,brain_) was determined using the rat brain slice-binding method (Pharmaron). The K_p,uu,brain_ (K_p,brain_ × f_u,brain_/f_u,plasma_) and K_p,u,CSF_ (K_p,CSF_/f_u,plasma_) values were then calculated.

### Efficacy tumor models

#### PDX tumor models

The LU0387 (Crown Bioscience) and LXFE2478 (Charles River Laboratories) tumors were harvested from the donor mice, and fragments 2 to 4 mm in diameter were inoculated subcutaneously in the right upper flank of the 6- to 8-week-old BALB/c nude (GemPharmatech, IMSR_GPT:D000521) and NMRI nu/nu female mice (Charles River Laboratories, RRID: IMSR_CRL:639), respectively. The animals were randomized into three groups (*n* = 10) when the mean tumor size reached approximately 150 mm^3^ (LU0387) or 50 to 250 mm^3^ (LXFE2478), and then orally dosed once daily with vehicle (PEG-400: 20% Captisol, 20:80 v/v) or BLU-451 at 10 or 50 mg/kg. After randomization, body weights were measured thrice weekly (LU0387) or daily (LXFE2478), and tumor volumes were measured thrice weekly (LU0387) or twice weekly (LXFE2478).

#### A769insASV Ba/F3 tumor model

The A769insASV Ba/F3 cells were grown in 10% FBS RPMI 1640 medium with 1× P/S until they reached approximately 70% to 80% confluency. The cells were resuspended in 1:1 RPMI:Matrigel at 1.25 × 10^7^ cells/mL, and 200 μL (2.5 × 10^6^ cells) was injected into the right flank of female SCID beige mice (Vivo Bio Tech). The animals were randomized into three groups (*n* = 9) when the mean tumor size reached approximately 150 mm^3^ and then orally dosed once daily with vehicle (PEG-400: 20% Captisol, 20:80 v/v) or BLU-451 at 10 or 50 mg/kg. After randomization, body weights and tumor volumes were measured three times per week.

#### G719S Ba/F3 tumor model

The G719S Ba/F3 cells were grown in 10% FBS RPMI 1640 medium and maintained at 0.1 × 10^6^ to 2 × 10^6^ cells/mL. The cells were resuspended in 1:1 Dulbecco’s PBS:Matrigel at 5 × 10^6^ cells/mL, and 200 μL (1 × 10^6^ cells) was injected into the right flank of female nonobese diabetic SCID mice (Charles River Laboratories, RRID: IMSR_CRL:394). The animals were randomized into three groups (*n* = 10) when the mean tumor size reached approximately 150 mm^3^ and then orally dosed once daily with vehicle (PEG-400: 20% Captisol, 20:80 v/v), BLU-451 at 25 or 50 mg/kg, osimertinib at 14 mg/kg, or afatinib at 7.5 mg/kg. After randomization, body weights and tumor volumes were measured two or three times per week.

For all subcutaneous tumor models, tumors were measured in two dimensions using a caliper, and the tumor volume was calculated using the formula volume = (length × width^2^)/2.

#### Intracranial PC9-Luc mouse tumor model

The 6- to 8-week-old female BALB/c nude mice (Beijing AniKeeper Biotech Co., Ltd.) were anesthetized by intramuscular injection of Zoletil 50 (Virbac). An incision of 0.5 cm was made along the skin over the midline to expose the coronal and sagittal suture junctions. PC9-Luc cells grown to subconfluence were harvested, and 2 × 10^5^ PC9-Luc cells suspended in 2 μL RPMI 1640 medium were injected into the right lateral ventricle, 1 mm lateral to the sagittal suture, 0.2 mm inferior to the coronal suture, with a precisely controlled 2.5-mm injection depth. The cells were slowly injected over a 1-minute period, and the needle was left in place for an additional minute before removing it. The injection site was sealed with bone wax, and the incision was closed. On day 9, mice were randomized into three groups (*n* = 10) based on body weight and total bioluminescence and then orally dosed once daily with vehicle (PEG-400: 20% Captisol, 20:80 v/v) or BLU-451 at 2.5 or 25 mg/kg. The body weights were measured three times per week throughout the study. The tumor burden was monitored twice weekly by imaging analysis as follows. Mice were injected intraperitoneally with 15 mg/mL of D-luciferin (Revvity) at 5 μL/g of body weight and anesthetized with isoflurane. Ten minutes later, mice were imaged using an IVIS Lumina II system (Revvity). Bioluminescence was quantified using Living Image software (Revvity). At the end of the study, whole brains and spinal cords were dissected and imaged, and their bioluminescence was quantified as described above.

#### Intracarotid PC9-Luc mouse tumor model

The 6- to 8-week-old female BALB/c nude mice (Shanghai Lingchang Biotechnology Co., Ltd.) were anesthetized by intraperitoneal injection of Zoletil 50 at 41.6 mg/kg, and an incision of 1 cm was made to expose the carotid artery. Loose sutures were placed distal and proximal to the injection site. After tightening the proximal knot to block the blood flow, PC9-Luc cells were grown to subconfluence, washed once and resuspended in PBS, and were injected into the carotid artery, and 100 μL containing 10^4^ PC9-Luc cells was injected per animal. The incision was then closed. On day 17, mice were randomized into six groups (*n* = 10) based on their bioluminescence and then orally dosed once daily with vehicle (PEG-400: 20% Captisol, 20:80 v/v in 100 mmol/L citrate buffer pH 3.5), BLU-451 (1, 2.5, 25 mg/kg), osimertinib (25 mg/kg), or erlotinib (15 mg/kg). Osimertinib and erlotinib were formulated in 20% Solutol HS 15 with 0.5% (w/v) sodium carboxymethyl cellulose. The body weights were measured twice weekly throughout the study. The tumor burden was monitored twice weekly by imaging analysis as follows. Mice were injected intraperitoneally with 15 mg/mL of D-luciferin (Revvity) in Dulbecco’s PBS at 10 μL/g of body weight and anesthetized with isoflurane. After 10 to 15 minutes, mice were imaged using an IVIS Lumina III system (Revvity). Bioluminescence was quantified using Living Image software (Revvity). At the end of the study, whole brains were dissected and imaged, and their bioluminescence was quantified as described above.

### Clinical study

The CONCERTO (NCT05241873) was a phase I/II, open-label, international, multicenter dose-escalation and -expansion trial of BLU-451, which was planned to evaluate BLU-451 as a single agent or in combination with platinum-based chemotherapy (carboplatin and pemetrexed) in adults with *EGFR-*mutated metastatic NSCLC. The primary objectives of the phase I portion of the study were to assess safety and tolerability and to identify the maximum tolerated dose and/or the recommended phase II dose of BLU-451 as monotherapy and in combination with platinum-based chemotherapy. The key eligibility criteria in the phase I monotherapy portion of the study included adults with Eastern Cooperative Oncology Group performance status of 0 to 1, adequate end-organ function, progression on or after intolerance to the most recent systemic therapy, and baseline evaluable or measurable disease by investigator assessment as per Response Evaluation Criteria in Solid Tumors (RECIST) version 1.1. Patients with CNS metastases were permitted if the brain disease was neurologically stable and did not require increasing corticosteroid doses. Prior platinum-based chemotherapy was required for patients with *EGFR* exon 20 insertion mutations, and prior treatment with ≥1 EGFR-TKI was required for patients with *EGFR* atypical mutations. Prior exon 20 insertion–targeted therapy was allowed, but not required, for patients with exon 20 insertion mutations. BLU-451 monotherapy was administered as 100-mg tablets to be taken either once daily or twice daily on an empty stomach or with food in 21-day cycles. Intrapatient dose escalation was permitted. The CONCERTO trial was approved by the institutional review board or independent ethics committee at each participating site and conducted in accordance with written standard operating procedures, the Declaration of Helsinki, and applicable global and local Good Clinical Practice and International Council for Harmonization guidelines. All patients gave written informed consent prior to study participation. The trial was discontinued in January 2024 because of sponsor decision.

### Plasma next-generation sequencing

Patient cycle 1 day 1 (baseline) and cycle 1 day 15 (on-treatment) whole-blood specimens were collected in Streck cell-free DNA (cfDNA) blood collection tubes. Using an established protocol generated and executed by Foundation Medicine Inc., the tubes were gently mixed by inversion and double-centrifuged to separate the plasma from the buffy coat and red blood cells. cfDNA was isolated and purified from plasma. Hybridization-based capture–selected libraries were sequenced using the Illumina NovaSeq platform, the sequence data were processed, and genomic alteration calls were reported using the custom analysis pipeline from Foundation Medicine Inc. Using established specimen and sequencing quality control standards, only paired patient specimens meeting all these necessary requirements were able to be used in downstream circulating tumor DNA (ctDNA) profiling analyses.

## Results

### Biochemical and cellular characterization of BLU-451 inhibitory potency

**Figure 1.**
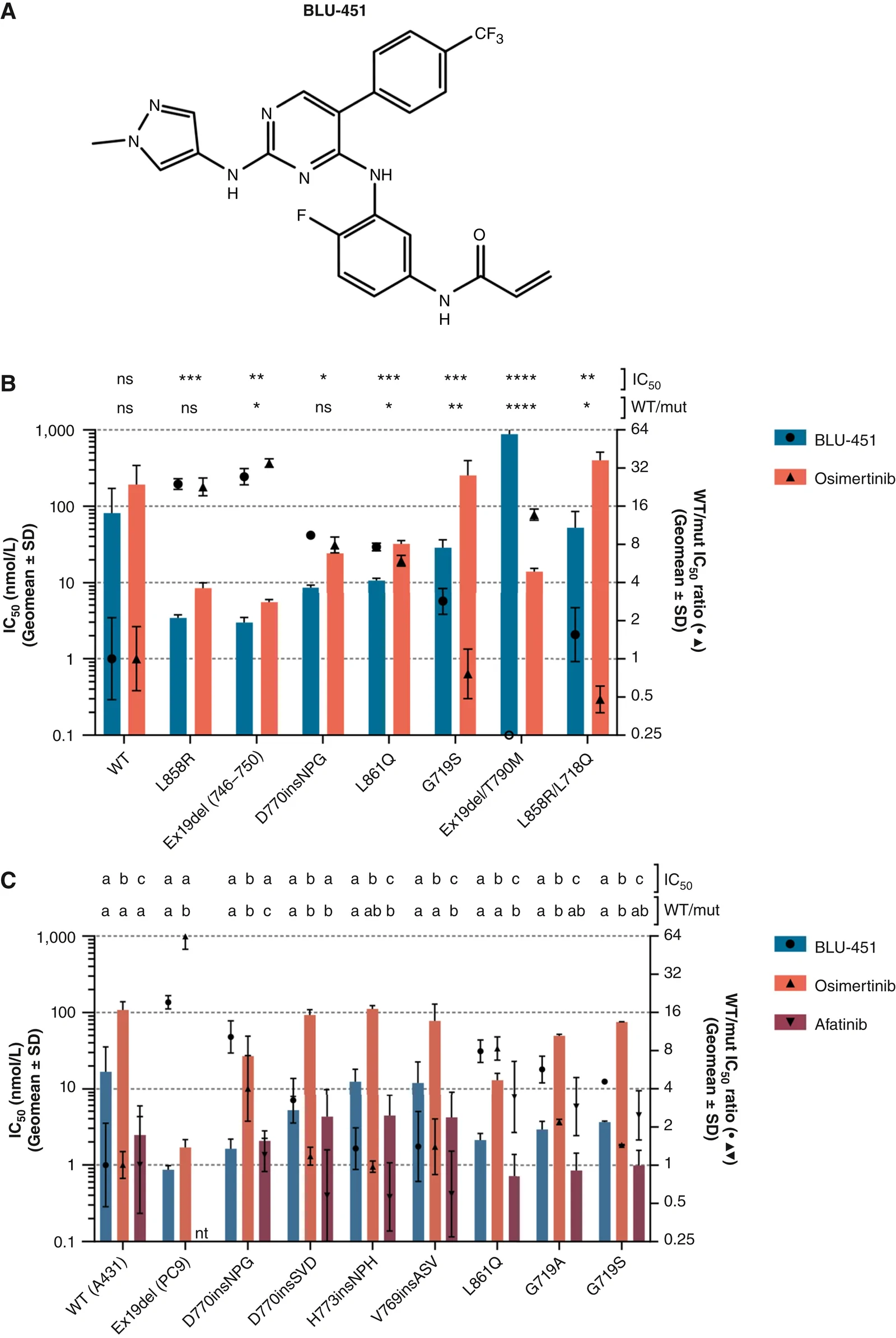
BLU-451 chemical structure and *in vitro* characterization of BLU-451, osimertinib, and afatinib inhibitory activity against *EGFR* variants. **A,** Chemical structure of BLU-451. **B,** Biochemical inhibition of the enzymatic activity of indicated *EGFR* variants by BLU-451 and osimertinib in the presence of 1 mmol/L of ATP. WT/mutant (mut) IC_50_ ratio for Ex19del/T790M (open circle) was 0.09. See “Materials and Methods” section for details. **C,** Cellular activity of BLU-451, osimertinib, and afatinib against EGFR phosphorylation. A431 (WT *EGFR*), PC9 (ex19del), or Ba/F3 cells expressing indicated *EGFR* variants were treated with BLU-451, osimertinib, or afatinib for 4 hours at 10 different doses. EGFR phosphorylation was then measured, and IC_50_ values were determined as described in the “Materials and Methods” section. Data represent the geometric mean (Geomean) value of at least two independent experiments ± SD; log-normal Welch’s *t* test (**A**); ns, not significant; *, *P* < 0.05; **, *P* < 0.01; ***, *P* < 0.001; ****, *P* < 0.0001; two-way ANOVA of log-transformed data with the Tukey multiple comparisons test (except for PC9, log-normal Welch’s *t* test; **B**); groups not connected by the same letter (a, b, and c) are significantly different from each other (*P* < 0.05).

The BLU-451 (Fig. 1A) was designed to be a WT *EGFR*–sparing, CNS-active, *EGFR* exon 20 insertion inhibitor. The core of BLU-451 consists of 2,4-bis-aminopyrimidine, with N1 and 2-NH forming hydrogen bonds to the hinge M793 residue of the EGFR ATP-binding site. The 2-NH group is substituted with methylpyrazole, which extends toward the solvent front. Fluoro-substituted phenyl acrylamide engages the target C797, and para-trifluoromethyl substituted phenyl is included at the 5-position on the pyrimidine to probe the gatekeeper pocket.

In recombinant EGFR enzymatic assays, BLU-451 showed potent inhibitory activity against common mutations (L858R and ex19del) and the D770insNPG exon 20 insertion, with low nanomolar IC_50_ (3.4, 3, and 8.6 nmol/L, respectively; Fig. 1B). BLU-451 also exhibited strong potency against atypical L861Q and G719S mutations, with the IC_50_ values of 11 and 29 nmol/L, respectively (Fig. 1B). Importantly, BLU-451 was significantly more active against those mutations compared with the WT EGFR protein (IC_50_ 81 nmol/L), with a fold selectivity ranging from ∼2.8× for G719S to ∼27× for the exon 19 deletion. Compared with osimertinib, BLU-451 showed improved potency and selectivity for L861Q and G719S and the osimertinib-resistant L858R/L718Q double mutant but was inactive against the secondary ex19del/T790M mutation, the major on-target acquired resistance to first-generation EGFR-TKIs (Fig. 1B). To assess its kinase selectivity profile, BLU-451 was tested at 1 μmol/L against 468 kinases (409 WTs and 59 mutants) in an active site-directed competitive binding assay (KINOMEscan). BLU-451 was highly selective, with only 1.7% of kinases showing >90% inhibition. The off-target screening hits were members of related ErbB family kinases (HER2 and HER4), kinases with a reactive cysteine (TEC, BTK, BLK, and TXK), and PAK3 (Supplementary Table S1; Supplementary Fig. S1). BLU-451 was also tested at 1 μmol/L against 370 kinases in an enzymatic activity assay in the presence of 10 μmol/L of ATP and found to only inhibit 2.2% of kinases with >90% inhibition, with all the off-target kinases having a reactive cysteine (Supplementary Table S2). Using both the screening methods, BLU-451 was found to be a highly selective kinase inhibitor with the potential for only a small number of off-target kinase effects.

**Figure 2.**
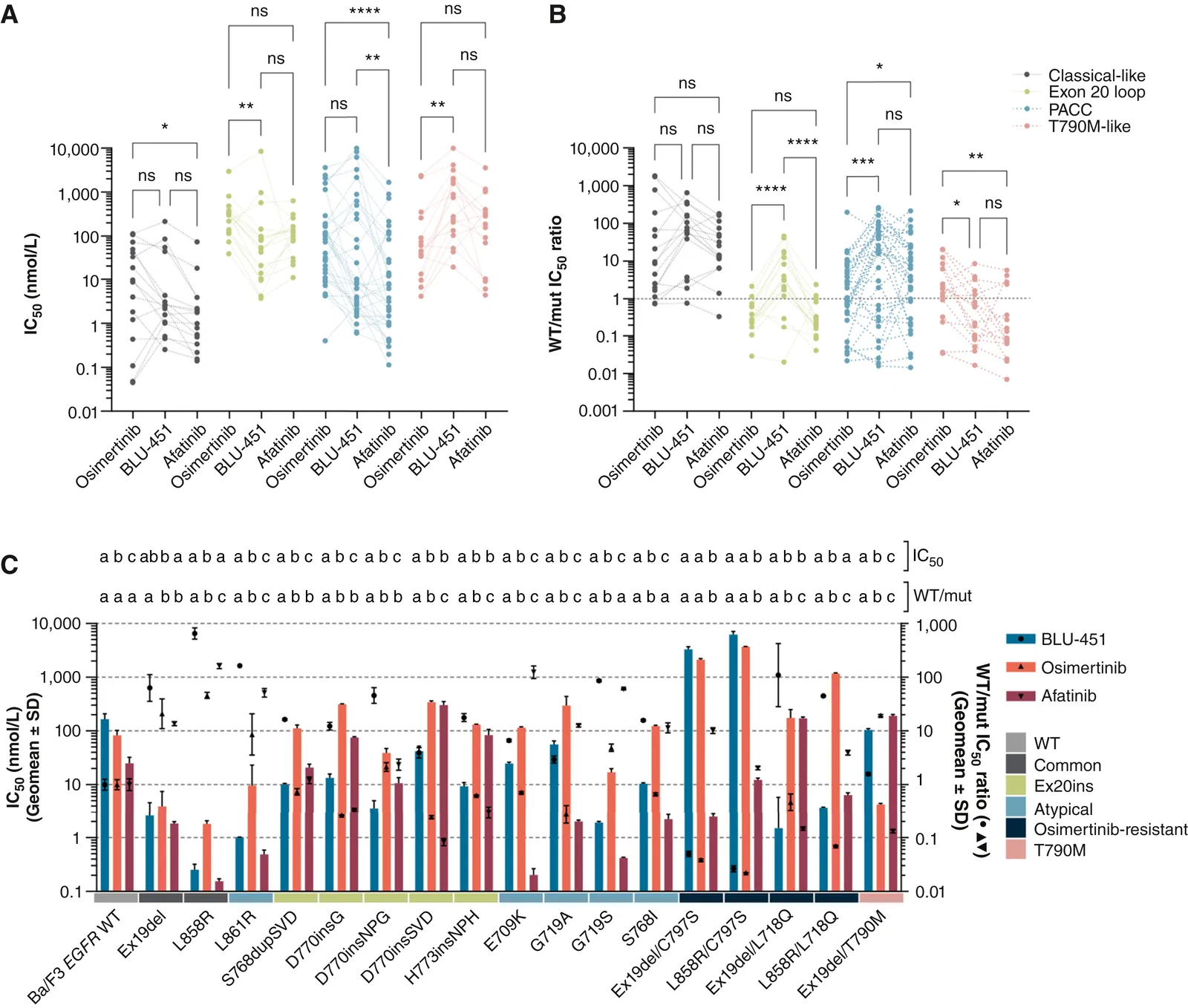
*In vitro* antiproliferative activity of BLU-451, osimertinib, and afatinib in Ba/F3 cell lines expressing EGFR from different subtypes. **A** and **B,** IC_50_ (**A**) and IC_50_ ratio [WT/mutant (mut); **B**] for Ba/F3 cells expressing *EGFR* variants from the classical-like, exon 20 loop insertion, PACC, and T790M-like subtypes (2) treated with BLU-451, osimertinib, or afatinib. The antiproliferation IC_50_ values for each cell line and treatment were determined as described in the “Materials and Methods” section. The WT/mut ratio was then calculated by dividing the IC_50_ values by IC_50_ obtained with the Ba/F3 cell line expressing WT *EGFR*. Data represent the geometric mean (Geomean) for each *EGFR* variant; log-normal one-way ANOVA data with the Tukey multiple comparisons test; ns, not significant; *, *P* < 0.05; **, *P* < 0.01; ***, *P* < 0.001; ****, *P* < 0.0001. **C,** Antiproliferation IC_50_ values and IC_50_ ratio (WT/mut) for BLU-451, osimertinib, and afatinib in Ba/F3 cells expressing WT and indicated *EGFR* variants. Data represent the geometric mean of *n* = 3 replicates; two-way ANOVA of log-transformed data with the Tukey multiple comparisons test. Groups not connected by the same letter (a, b, and c) are significantly different from each other (*P* < 0.05). Ex20ins, exon 20 insertion.

BLU-451, osimertinib, and afatinib cellular activity against EGFR phosphorylation was evaluated in engineered Ba/F3 cell lines expressing exon 20 insertions or atypical mutations and the human NSCLC PC9 cell line with an exon 19 deletion (Fig. 1C). Consistent with the biochemical data, BLU-451 showed strong inhibitory activity against the ex19del, D770insNPG, L861Q, G719A, and G719S mutants with IC_50_ values of 0.9, 1.6, 2.1, 2.9, and 3.7 nmol/L, respectively. BLU-451 was also potent against D770insSVD (5.2 nmol/L), H773insNPH (12.5 nmol/L), and V769insASV (12 nmol/L). In all these cell lines, BLU-451 showed greater potency compared with the WT EGFR cell line A431. Compared with osimertinib, BLU-451 improved potency for all exon 20 insertions and atypical mutations, ranging from 6-fold for L861Q to 16-fold for D770insNPG. BLU-451 also improved selectivity over WT EGFR by ∼3- to 4-fold for all those uncommon mutations except H773insNPH, V769insASV, and L861Q, both showing similar WT/mutant IC_50_ ratios between BLU-451 and osimertinib. Compared with afatinib, BLU-451 improved selectivity over WT EGFR for all the cell lines except for G719A and G719S. BLU-451 activity against cellular proliferation was then tested in engineered Ba/F3 cell lines expressing WT EGFR and 81 EGFR kinase domain mutations belonging to the classical-like, exon 20 loop insertions, PACC and T790M-like structure-based subsets, alongside osimertinib and afatinib (2). BLU-451 exhibited antiproliferative activity against a wide range of *EGFR* mutations belonging to different subsets (Fig. 2A; Supplementary Fig. S2A). In particular, BLU-451 showed superior potency and selectivity for exon 20 loop insertions overall compared with osimertinib and afatinib (Fig. 2A and B; Supplementary Fig. S2). Importantly, BLU-451 was active against some of the most prevalent exon 20 insertions (S768dupSVD, D770insG, D770insNPG, D770insSVD, and H773insNPH) and atypical mutations (E709K, G719X, L861X, and S768I), as well as the common mutations L858R and exon 19 deletions, with greater potency over WT *EGFR* (Fig. 2C; Supplementary Fig. S2). As expected for a covalent inhibitor, BLU-451 showed poor activity against the osimertinib-resistant ex19del/C797S and L858R/C797S compound mutations. However, BLU-451 was potent and selective for the osimertinib-resistant ex19del/L718Q and L858R/L718Q compound mutations, consistent with the biochemical data (Fig. 2C; Supplementary Fig. S2). Osimertinib showed stronger potency against the ex19del/T790M and L858R/T790M mutations compared with BLU-451 and afatinib, which is expected for a third-generation TKI (Fig. 2C; Supplementary Fig. S2).

### Evaluation of BLU-451 efficacy in tumor models of uncommon *EGFR* mutations

**Figure 3.**
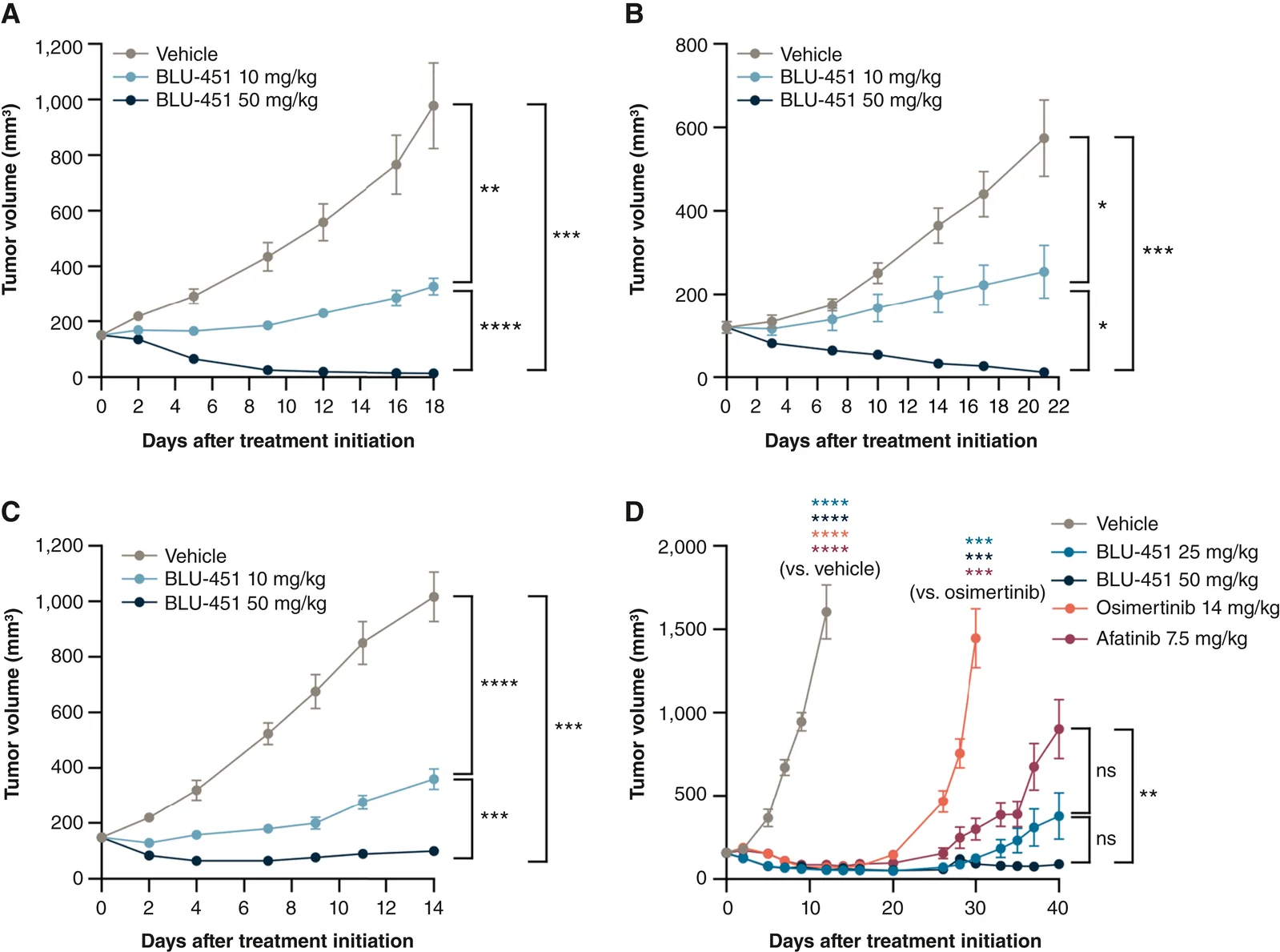
*In vivo* antitumor activity of BLU-451 in uncommon EGFR PDX and Ba/F3 mouse tumor models. **A,** Tumor growth in the LU0387 PDX model carrying the *EGFR* exon 20 insertion H773insNPH. **B,** Tumor growth in the LXFE2478 PDX model carrying the *EGFR* exon 20 insertion V769insASV. **C,** Tumor growth in the *EGFR* exon 20 insertion V769insASV Ba/F3 tumor model. **D,** Tumor growth in the *EGFR* G719S Ba/F3 tumor model. Mice were orally dosed once daily with BLU-451 at 10, 25, or 50 mg/kg, osimertinib at 14 mg/kg, and afatinib at 7.5 mg/kg as indicated. Data represent the average ± SEM of 10 (**A**, **B**, and **D**) or 9 (**C**) animals per group; Brown–Forsythe and Welch ANOVA with the Dunnett T3 multiple comparisons test; ns, not significant; *, *P* < 0.05; **, *P* < 0.01; ***, *P* < 0.001; ****, *P* < 0.0001.

The BLU-451 *in vivo* antitumor activity was evaluated in *EGFR* exon 20 insertion PDX models: LU0387 (H773insNPH) and LXFE2478 (V769insASV; Fig. 3A and B). In both the models, once-daily oral administration of BLU-451 led to marked, dose-dependent antitumor activity. At 50 mg/kg BLU-451, tumor regression (defined as a >30% decrease in tumor size compared with baseline) was observed in all treated animals in both the models, and complete tumor regression was achieved in 6 of 10 animals in the LU0387 model. In the LU0387 model, the 50 mg/kg dose resulted in 91% tumor regression at day 18, whereas 90% tumor regression was observed at day 21 in the LXFE2478 model. BLU-451 antitumor activity was also evaluated in the V769insASV and G719S Ba/F3 subcutaneous mouse tumor models (Fig. 3C and D). At the 50 mg/kg dose, tumor regression was achieved in six of nine animals for V769insASV and 8 of 10 animals for G719S. In the V769insASV Ba/F3 model, dosing with 50 mg/kg resulted in 34% tumor regression at day 14. In the G719S Ba/F3 model, BLU-451 25 and 50 mg/kg doses led to 60% tumor regression at day 12. Although similar tumor regression was observed with 14 mg/kg osimertinib and 7.5 mg/kg afatinib at day 12, BLU-451 treatment led to a longer duration of response. The waterfall plots showing antitumor activity of BLU-451 in all mouse tumor models are shown in Supplementary Fig. S3. In all studies, BLU-451 was well tolerated, with body weight loss never exceeding 10% (Supplementary Fig. S4).

### PK/PD evaluation of BLU-451 in tumor, large intestine, and skin tissues

**Figure 4.**
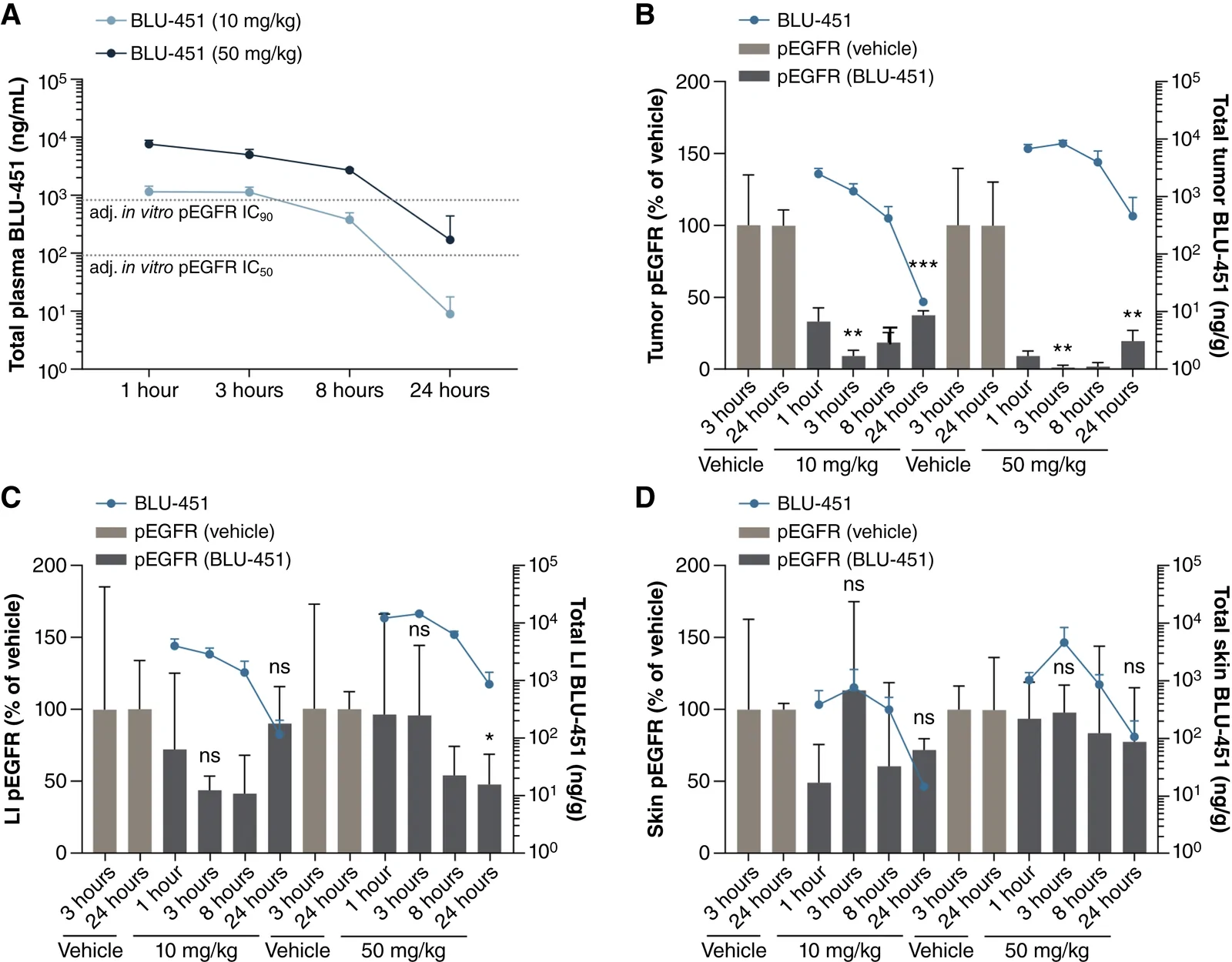
PK/PD relationship of BLU-451 in the *EGFR* exon 20 insertion V769insASV Ba/F3 mouse tumor model. **A,** BLU-451 total plasma levels after a single oral dose of BLU-451 at 10 or 50 mg/kg in the V769insASV Ba/F3 mouse tumor model. Dotted lines indicate the BLU-451 IC_50_ and IC_90_ values adjusted for plasma protein binding from the *in vitro* EGFR phosphorylation assay in V769insASV Ba/F3 cells. **B–D,** BLU-451 total levels and pEGFR normalized to vehicle control in tumor (**B**), large intestine (LI; **C**), and skin (**D**) in the V769insASV Ba/F3 mouse tumor model. pEGFR (Tyr1068) was determined using Western blot analysis and BLU-451 levels by LC/MS-MS. Data represent the average of three animals ± SD. Statistical analysis: comparison with vehicle; one-way ANOVA with Tukey multiple comparisons test; ns, not significant; *, *P* < 0.05; **, *P* < 0.01; ***, *P* < 0.001.

The relationship between BLU-451 concentrations and pEGFR was analyzed in the Ba/F3 V769insASV subcutaneous mouse tumor model (Fig. 4). A single oral administration of BLU-451 at 10 and 50 mg/kg led to a dose-dependent increase in plasma drug concentration that covered the V769insASV cellular pEGFR IC_50_ (adjusted for plasma protein binding) for more than 8 and 24 hours, and the 90% of maximal value for inhibitory concentration (IC_90_) for more than 3 and 8 hours, respectively (Fig. 4A). Concordantly, pEGFR was significantly suppressed in tumors in a dose- and time-dependent manner, with the 50 mg/kg dose leading to a near-complete suppression at the 3- and 8-hour time points (Fig. 4B). Despite BLU-451 reaching similar exposure in tumors and the large intestine, BLU-451 did not significantly suppress pEGFR in the large intestine except at the 24-hour time points with the 50 mg/kg dose (Fig. 4C). The lack of correlation between BLU-451 exposure and pEGFR inhibition in the large intestine suggests that this inhibition was not compound related. Additionally, BLU-451 did not block pEGFR in the skin tissue at levels that significantly inhibit pEGFR in tumors (Fig. 4D). Taken together, these results demonstrate that BLU-451 can strongly suppress exon 20 insertion pEGFR expression levels *in vivo* while sparing WT EGFR. The Western blots used to quantify pEGFR in tumor, skin, and large intestine tissues are shown in Supplementary Fig. S5.

### Evaluation of BLU-451 brain penetration and intracranial efficacy

**Figure 5.**
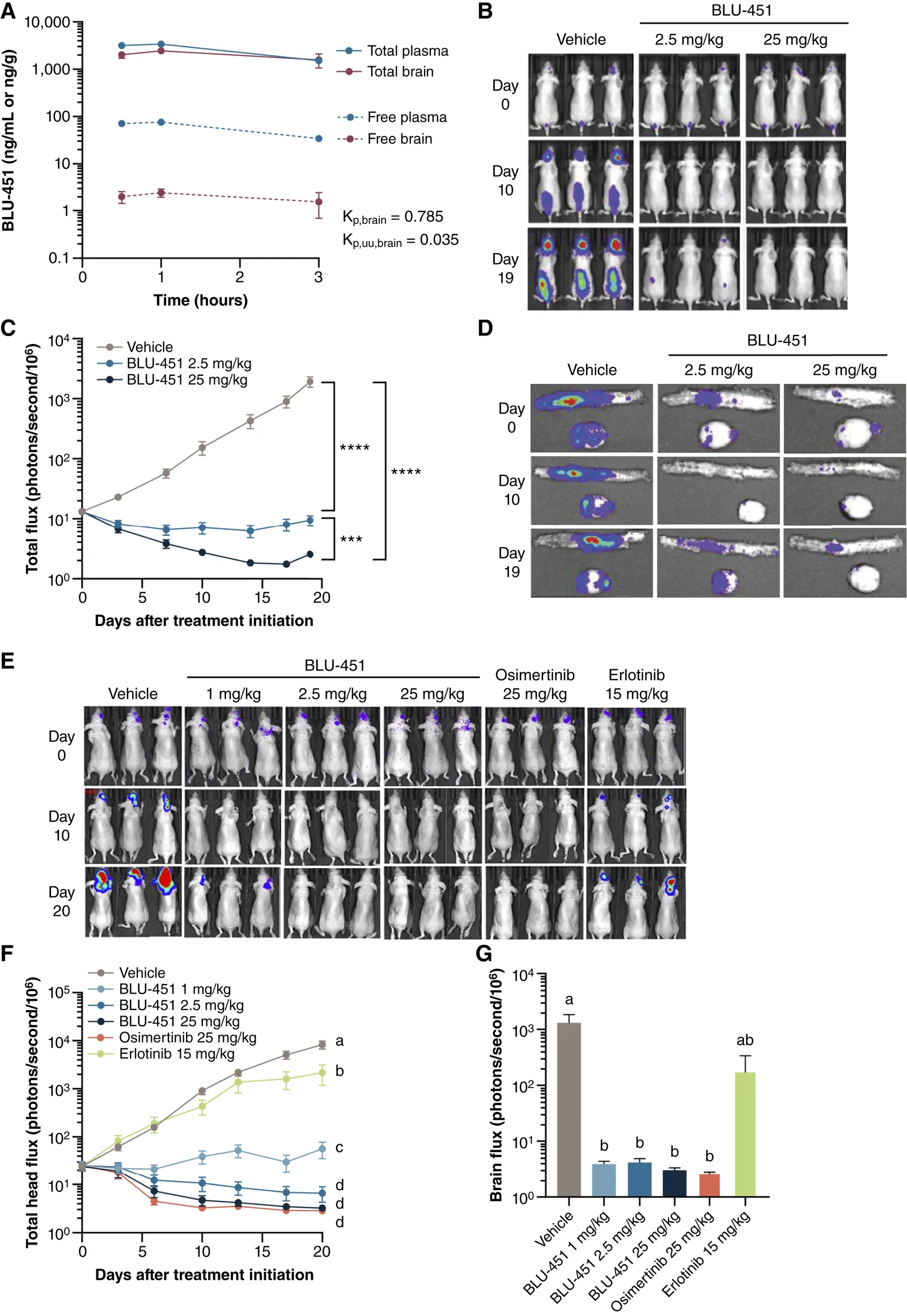
BLU-451 brain penetration and intracranial efficacy. **A,** Total and free BLU-451 plasma and brain levels at 0.5 hour, 1 hour, and 3 hours after a single oral dose of BLU-451 at 20 mg/kg dose in BALB/c mice. Data represent the average of three animals ± SEM. The K_p,brain_ and K_p,uu,brain_ values were determined as described in the “Methods” section. **B–D,** PC9-Luc intracranial injection model in BALB/c nude mice orally dosed once daily with BLU-451 at 2.5 or 25 mg/kg. Visualization of whole-body tumor burden at days 0, 10, and 19 after treatment initiation (**B**). Total bioluminescence after treatment initiation (**C**). Visualization of tumor burden in dissected spinal cord (top) and brain (bottom) at day 19 after treatment initiation (**D**). **E–G,** PC9-Luc intracarotid injection model in BALB/c nude mice orally dosed once daily with BLU-451 at 1, 2.5, or 25 mg/kg, osimertinib at 25 mg/kg, or erlotinib at 15 mg/kg. Visualization of whole-body tumor burden at days 0, 10, and 20 after treatment initiation (**E**). Total head bioluminescence after treatment initiation (**F**). Bioluminescence in dissected brain at day 20 after treatment initiation (**G**). Images in **B**, **D**, and **E** are representative of 10 animals. Images for all animals are shown in Supplementary Figs. S7 and S8. Data in **C**, **F**, and **G** represent the average of 10 animals ± SEM; log-normal Brown–Forsythe and Welch ANOVA with the Dunnett T3 multiple comparisons test; ***, *P* < 0.001; ****, *P* < 0.0001. Groups not connected by the same letter (a, b, c, and d) are significantly different from each other (*P* < 0.05). K_p,brain_, AUC_brain_/AUC_plasma_; K_p,uu,brain_, K_p,brain_ × f_u,brain_/f_u,plasma_.

In order to evaluate its potential for brain penetration *in vitro*, BLU-451 was tested in MDR1- and BCRP-overexpressing MDCKII cell permeability assays. BLU-451 was found to be a weak substrate for those efflux transporters, with the efflux ratios of 1.6 and 2 for MDR1 and BCRP, respectively. A single oral dose of BLU-451 at 30 mg/kg to Wistar Han rats led to equal plasma and brain total exposure with a brain-to-plasma ratio (K_p,brain_) of 1.02 (Supplementary Fig. S6A). In rats dosed intravenously with BLU-451 at 20 mg/kg by continuous infusion, K_p,brain_ was 0.61 (Supplementary Fig. S6B). BLU-451 was highly bound to rat brain tissue *in vitro* (f_u,brain_ of 0.001 using the rat brain-slice method), which resulted in an unbound brain-to-plasma ratio K_p,uu,brain_ of 0.026 and 0.016 when dosed orally or by continuous infusion, respectively (Supplementary Fig. S6). In both studies, BLU-451 levels in the CSF exceeded the cellular pEGFR IC_50_ values of BLU-451 against EGFR exon 20 insertions and atypical mutations with target coverage ranging from 4-fold for V769insASV to 60-fold for L861Q. The observed K_p,u,CSF_ for BLU-451 was 0.86 and 0.46 when dosed orally or by continuous infusion, respectively. Following a single oral dose of BLU-451 at 20 mg/kg to BALB/c mice, BLU-451 achieved similar total exposure in brain and plasma, with a K_p,brain_ of 0.785 (Fig. 5A). Using the rat f_u,brain_ value, the calculated K_p,uu,brain_ in mice was 0.035. In a similar study, comparable values were obtained after 5 days of 10 and 50 mg/kg once daily oral administrations at 4 and 12 hours after dosing (Supplementary Fig. S7).

The potential for BLU-451 in metastatic brain disease was next explored in a mouse model, in which PC9-Luc was intracranially injected to simulate the clinical condition. BLU-451 at 5 and 25 mg/kg resulted in tumor shrinkage of 29% and 80%, respectively, and led to >99% reduction in total bioluminescence signal compared with vehicle for both doses (Fig. 5B and C). This result was confirmed by terminal *ex vivo* imaging analysis of the brain and spinal cord showing a dose-dependent decrease in bioluminescence signal in BLU-451–treated mice relative to control mice (Fig. 5D). BLU-451 was then evaluated in a PC9-Luc intracarotid injection in a mouse model, alongside osimertinib and erlotinib. Similar to the intracranial injection model, BLU-451 led to tumor regression and a dose-dependent reduction of the head bioluminescence signal compared with vehicle (Fig. 5E and F). BLU-451 at 2.5 and 25 mg/kg resulted in tumor shrinkage of 73% and 87%, respectively, and >99.9% reduction in total bioluminescence signal compared with vehicle. Consistent with its potency against exon 19 EGFR deletion, brain penetration, and CNS activity (23–25), osimertinib treatment also led to 89% tumor regression and complete reduction in total bioluminescence compared with vehicle (>99.9%), unlike erlotinib, which only led to partial tumor growth inhibition (74%). These results were further confirmed by terminal *ex vivo* analysis of the brain bioluminescence (Fig. 5G). All images used for quantification are shown in Supplementary Figs. S8 and S9. Taken together, these studies show that BLU-451 has the potential for targeting CNS disease.

### First-in-human clinical activity of BLU-451 monotherapy in patients with metastatic NSCLC harboring *EGFR* exon 20 insertions and atypical mutations

**Figure 6.**
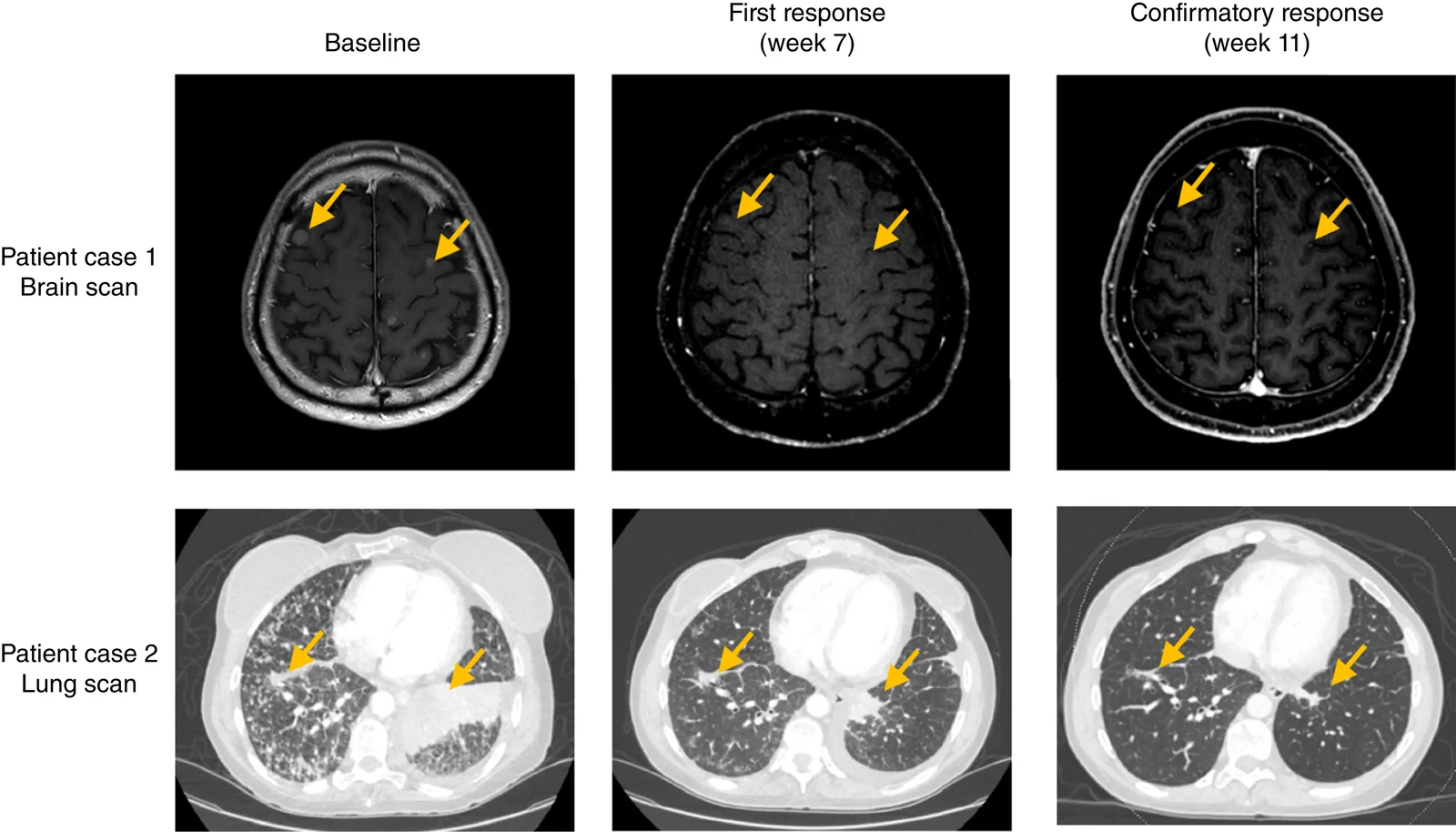
BLU-451 demonstrates early clinical activity. Images of target lesions in the brain (patient case 1) and lung (patient case 2) are shown at baseline, first response at 7 weeks, and confirmatory response at 11 weeks.

The antitumor activity of BLU-451 was assessed in a first-in-human phase I/II clinical trial of BLU-451 (CONCERTO, NCT05241873). The trial was initiated in March 2022 and was designed to assess the safety, tolerability, and efficacy of BLU-451 as a single agent and in combination with platinum-based chemotherapy in patients with advanced NSCLC with *EGFR* exon 20 insertion or atypical mutations. Two examples of early overall and CNS antitumor activity with BLU-451 in patients harboring *EGFR* exon 20 insertions and atypical mutations from the phase I dose-escalation BLU-451 monotherapy portion of the study are described here (Fig. 6).

#### Case 1

The patient was a 46-year-old Asian male with stage IVB NSCLC (adenocarcinoma) with *EGFR* exon 20 insertion mutation, *EGFR* H773insNPH. Local tissue next-generation sequencing (NGS)–based molecular testing at screening also identified co-occurring *EGFR* amplification and p63 mutations, *TP63* A159P and *TP63* R266* (a nonsense mutation that causes premature truncation of the p63 protein at amino acid 266). Prior systemic therapy in the metastatic setting included carboplatin and pemetrexed with the best overall response (BOR) of stable disease, followed by mobocertinib (approved at the time of use for patients with *EGFR* exon 20 insertion mutation–positive locally advanced or metastatic NSCLC; withdrawn from use in 2023) with the BOR of partial response (PR). Upon progression on mobocertinib, and after the required washout period per the study protocol, the patient was enrolled into the phase I BLU-451 monotherapy portion of the CONCERTO study. At baseline, the patient had one target lesion in the adrenal gland and two previously untreated target lesions in the brain, in addition to nontarget lesions in the lung, brain, and bone. The patient received BLU-451 at a dose of 400 mg twice daily, continuously in 21-day cycles. Disease assessment at 7 weeks of treatment showed an overall and CNS PR as per RECIST version 1.1, with −40% change in the sum of the longest diameter of the target lesions overall and −67% change in the sum of the longest diameter of the target lesions in brain, respectively, compared with baseline. In addition, nontarget brain lesions showed complete response. CNS responses (Fig. 6) and overall responses were confirmed on the subsequent imaging at 11 weeks of treatment (Fig. 6). The patient tolerated treatment well, with no observed grade ≥3 AEs. The patient maintained the overall and CNS responses up to week 19 before discontinuing treatment due to a clinical progression at week 23.

#### Case 2

The patient was a 53-year-old White female with stage IVB NSCLC (adenocarcinoma) with *EGFR* exon 20 insertion mutation, *EGFR* D770insY. Local plasma NGS-based molecular testing at screening also identified a co-occurring Janus kinase 2 V617F mutation. Prior systemic therapy in the metastatic setting included carboplatin and pemetrexed with the BOR of progressive disease. Upon progression on platinum-based chemotherapy and after the required washout period per study protocol, the patient was enrolled into the phase I BLU-451 monotherapy portion of the CONCERTO study. At baseline, the patient had two target lesions in the lung in addition to multiple nontarget lesions in the lung and bone. The patient received BLU-451 initially at a dose of 200 mg twice daily, followed by 400 mg once daily at 16 weeks and then reduced to 300 mg once daily at 18 weeks due to a grade II total bilirubin increase. Disease assessment at 7 weeks of treatment showed a PR as per RECIST version 1.1, with −46% change in the sum of the longest diameter of the target lesions. The PR was confirmed on subsequent imaging at 11 weeks of treatment with further tumor shrinkage of −54% compared with baseline, and the sum of the measurements continued to decrease to −65% change from baseline. The patient tolerated treatment well, with no observed grade ≥3 AEs. The patient maintained the response (Fig. 6) up to week 49 and at the time of publication remains on therapy. Cycle 1 day 15 results from ctDNA profiling indicated complete ctDNA clearance of *EGFR* D770_N771insY compared with the paired baseline level (Supplementary Fig. S10).

Aligned with the observed clinical responses, steady-state free BLU-451 plasma levels after 15 days of dosing exceeded the *in vitro* antiproliferative IC_90_ (Ba/F3) for the H773insNPH and D770insX EGFR exon 20 insertions corrected for FBS binding, with C_min,ss,u_ of 2.85 and 2 ng/mL for cases 1 and 2, respectively (Supplementary Table S4). In both patients, free plasma exposures were also comparable to those associated with antitumor efficacy in mouse models (Supplementary Tables S3 and S4).

## Discussion

For patients with uncommon *EGFR* mutations, including exon 20 insertions and atypical mutations, treatment options remain limited. For patients with exon 20 insertions, amivantamab, a bispecific EGFR and cMET antibody, in combination with platinum-based chemotherapy, is the current and only standard of care in the first-line setting; however, this combination is associated with significant toxicity, and amivantamab is predicted to have limited CNS penetration (14, 26). Although sunvozertinib, a TKI targeting exon 20 insertions, has recently received accelerated approval for patients who have progressed on or after platinum-based chemotherapy, this drug has also significant AEs, including rash, ocular toxicity, interstitial lung disease, and gastrointestinal toxicities (27). For patients with *EGFR* atypical mutations, only the second-generation EGFR-TKI afatinib is approved for certain atypical mutations but is often poorly tolerated because of off-target effects on WT EGFR (16, 28), and therefore osimertinib is frequently used off-label in this patient population (29). Therefore, the need for more selective and tolerable TKIs that can improve treatment outcomes in patients with uncommon *EGFR* mutations remains.

In this study, we describe the development, characterization, and early clinical experience of BLU-451, a novel CNS-active, potent, selective, and WT-sparing inhibitor of uncommon *EGFR*-mutant NSCLC. Biochemical and cellular profiling demonstrated the potent preclinical activity of BLU-451 against exon 20 insertions and atypical mutated EGFR with notable selectivity over WT EGFR. BLU-451 also exhibited significant antiproliferative activity in Ba/F3 cells expressing these mutations, showing improved selectivity over WT EGFR for the most common exon 20 insertion mutations compared with osimertinib and afatinib (Fig. 2). These data suggest that BLU-451 could potentially have lower WT EGFR toxicity, leading to a preferable safety profile. The molecular basis for BLU-451 selectivity for mutant over WT EGFR is not fully understood and, although it is outside the scope of this article, represents an interesting area of future study.

The BLU-451 showed an extended PD half-life, potently suppressing pEGFR expression in Ba/F3 V769insASV subcutaneous mouse tumors (Fig. 4), likely due to its covalent mechanism of action, and we demonstrated that BLU-451 has strong antitumor activity *in vivo*, with marked tumor regression in PDX models with *EGFR* exon 20 insertion mutations (Fig. 3). BLU-451 also showed comparable efficacy with osimertinib in the intracarotid injection brain metastasis mouse model. Despite the limitation that this model used PC9 cells carrying a common *EGFR* mutation rather than exon 20 insertions or atypical mutations, these results provide proof of concept that BLU-451 can be efficacious in the CNS and are promising given the limited treatment options available for patients with exon 20 insertion mutations. Notably, we found that BLU-451 unbound brain-to-plasma ratio (K_p,uu,brain_) in rats was lower than what has been reported for osimertinib (0.026 vs. 0.21) when using a comparable methodology, but superior to the poorly brain-penetrant afatinib (0.0062; ref. 24). Unlike BLU-451, erlotinib treatment did not lead to tumor regression and only partially inhibited tumor growth in the intracarotid injection model (Fig. 5) despite a reported K_p,uu,brain_ higher than BLU-451 in rats (0.084 vs. 0.026; ref. 24), supporting other evidence that K_p,uu,brain_ does not necessarily correlate with PD effects and CNS outcomes (24, 30, 31). For example, the TKI adagrasib, approved for the treatment of advanced or metastatic *KRAS* G12C–mutated NSCLC and CRC, demonstrated intracranial activity (32, 33) despite us finding it to be highly bound to brain tissue with a calculated K_p,uu,brain_ of ∼0.008 in mice based on a reported study (34). Similarly, sunvozertinib, although its clinical efficacy against brain metastases is yet to be fully characterized, showed antitumor activity in patients with brain metastases (35) despite a reported K_p,uu,brain_ of 0.008 (36). Those results indicate that brain penetration (often defined as K_p,uu,brain _ >0.3) is not necessary to achieve CNS responses, possibly due to an increased permeability of the blood–brain barrier in the context of metastatic lung cancer (37). Therefore, BLU-451 preclinical efficacy in the CNS has the potential to translate into humans, despite a low K_p,uu,brain_ relative to osimertinib (24). Importantly, BLU-451 penetrated the CSF to a greater extent than other EGFR-TKIs, including osimertinib (K_p,u,CSF_ of 0.86 vs. 0.29; ref. 24). This result highlights the potential of BLU-451 for the treatment of leptomeningeal disease affecting 11% of patients with advanced EGFR-mutated NSCLC at the time of metastatic diagnosis (38).

Early clinical cases support our preclinical findings. BLU-451 reduced the size of overall lesions as well as CNS lesions in pretreated patients with exon 20 insertions or atypical *EGFR* mutations. ctDNA profiling revealed complete ctDNA clearance in a patient with NSCLC bearing *EGFR* exon 20 insertion mutation at cycle 1 day 15 (Supplementary Fig. S10). These data are encouraging, considering the need for EGFR exon 20–targeted therapies with both improved efficacy and safety profiles. However, the safety profile and PK properties of BLU-451 require further evaluation to fully understand its potential adverse effects and whether an optimal dosing regimen can be achieved.

Despite the combination of amivantamab and chemotherapy showing improved treatment outcomes compared with chemotherapy alone, its predicted limitations in penetrating the blood–brain barrier (39–41) and AEs, including hematologic and skin-related EGFR toxicities (14, 39), highlight the ongoing need for potent, well-tolerated oral therapies that can be active in the CNS. Preclinical studies have demonstrated BLU-451 as a potent inhibitor of exon 20 insertions and atypical mutated *EGFR*, with antitumor efficacy in the CNS. Clinical activity with BLU-451, including tumor shrinkage, was observed in multiple patients, with early signs of intracranial activity, a key differentiator for this compound. However, due to the early-phase nature of the study and its early termination following business reprioritization, the overall response rate and durability could not be fully characterized. Although the CONCERTO trial has been discontinued from clinical development, these findings support further investigation of BLU-451 or other CNS-active EGFR inhibitors to better define their clinical benefit.

## Supplementary Material

Supplementary Tables Supplementary Tables S1-S4

Figure S1 Kinome tree showing the kinase selectivity of BLU451

Figure S2 BLU451 potency and selectivity against BaF3 cells expressing EGFR variants from different subtypes

Figure S3 Effect of BLU451 treatment on tumor growth in uncommon EGFR PDX and BaF3 mouse tumor models

Figure S4 Effect of BLU451 treatment on body weight in in vivo tumor models

Figure S5 Western blot analysis of EGFR phosphorylation in the A769insASV BaF3 mouse tumor model

Figure S6 Pharmacokinetics of plasma BLU451 after an oral dose of BLU451 at 30 mgkg or intravenous continuous infusion of BLU451 at 20 mgkg in 7

Figure S7 BLU451 pharmacokinetic analysis in BALBc mice after repeated dosing

Figure S8 Images of the PC9luciferase intracranial model showing whole-body images and ex vivo images of spinal cord and brain samples

Figure S9 Wholebody images of the PC9luciferase intracarotid model

Figure S10 ctDNA profiling in a patient with stage IVB NSCLC with EGFR exon 20 insertion mutation

## Data Availability

The data generated in this study are available within the article and supplementary data files.

## References

[1] Riess JW , Gandara DR, Frampton GM, Madison R, Peled N, Bufill JA, . Diverse EGFR exon 20 insertions and co-occurring molecular alterations identified by comprehensive genomic profiling of NSCLC. J Thorac Oncol 2018;13:1560–8. 10.1016/j.jtho.2018.06.019 PMC6764748 29981927

[2] Robichaux JP , Le X, Vijayan RSK, Hicks JK, Heeke S, Elamin YY, . Structure-based classification predicts drug response in EGFR-mutant NSCLC. Nature 2021;597:732–7. 10.1038/s41586-021-03898-1 PMC8481125 34526717

[3] Russo A , Franchina T, Ricciardi G, Battaglia A, Picciotto M, Adamo V. Heterogeneous responses to epidermal growth factor receptor (EGFR) tyrosine kinase inhibitors (TKIs) in patients with uncommon EGFR mutations: new insights and future perspectives in this complex clinical scenario. Int J Mol Sci 2019;20:1431. 10.3390/ijms20061431 PMC6470640 30901844

[4] Bai R , Chen X, Song W, Tian H, Cui J. Therapeutic exploration of uncommon EGFR exon 20 insertion mutations in advanced non-small cell lung cancer: breaking through brambles and thorns. J Cancer Res Clin Oncol 2022;148:163–76. 10.1007/s00432-021-03840-y PMC11801059 34698913

[5] Borgeaud M , Parikh K, Banna GL, Kim F, Olivier T, Le X, . Unveiling the landscape of uncommon EGFR mutations in NSCLC-a systematic review. J Thorac Oncol 2024;19:973–83. 10.1016/j.jtho.2024.03.016 38499147

[6] Leal JL , Alexander M, Itchins M, Wright GM, Kao S, Hughes BGM, . EGFR exon 20 insertion mutations: clinicopathological characteristics and treatment outcomes in advanced non-small cell lung cancer. Clin Lung Cancer 2021;22:e859–69. 10.1016/j.cllc.2021.04.009 34127383

[7] Li L . Two non-small cell lung cancer (NSCLC) patients with brain metastasis harboring epidermal growth factor receptor (EGFR) G719X and L861Q mutations benefited from aumolertinib: two cases report and review of the literature. Heliyon 2022;8:e10407. 10.1016/j.heliyon.2022.e10407 PMC9474834 36119883

[8] Ullas B , Shrinidhi N, Mansi S, Narayan S, Parveen J, Surender D, . All EGFR mutations are (not) created equal: focus on uncommon EGFR mutations. J Cancer Res Clin Oncol 2023;149:1541–9. 10.1007/s00432-022-04033-x PMC11796488 35581383

[9] Yang G , Li J, Xu H, Yang Y, Yang L, Xu F, . EGFR exon 20 insertion mutations in Chinese advanced non-small cell lung cancer patients: molecular heterogeneity and treatment outcome from nationwide real-world study. Lung Cancer 2020;145:186–94. 10.1016/j.lungcan.2020.03.014 32336530

[10] Olivier T , Prasad V. Amivantamab and mobocertinib in exon 20 insertions EGFR mutant lung cancer, challenge to the current guidelines. Transl Oncol 2022;23:101475. 10.1016/j.tranon.2022.101475 PMC9256830 35785671

[11] Park K , Haura EB, Leighl NB, Mitchell P, Shu CA, Girard N, . Amivantamab in EGFR exon 20 insertion-mutated non-small-cell lung cancer progressing on platinum chemotherapy: initial results from the CHRYSALIS phase I study. J Clin Oncol 2021;39:3391–402. 10.1200/JCO.21.00662 PMC8791812 34339292

[12] U.S. Food and Drug Administration . FDA approves amivantamab-vmjw for EGFR exon 20 insertion-mutated non-small cell lung cancer indications. Silver Spring (MD): U.S. Food and Drug Administration; 2024 [cited 2024 Sept 25]. Available from: https://www.fda.gov/drugs/resources-information-approved-drugs/fda-approves-amivantamab-vmjw-egfr-exon-20-insertion-mutated-non-small-cell-lung-cancer-indications.

[13] National Comprehensive Cancer Network . NCCN clinical practice guidelines in oncology (NCCN Guidelines®): non-small cell lung cancer. Version 8.2024. Plymouth Meeting (PA): National Comprehensive Cancer Network; 2024 [cited 2024 Sept 25]. Available from: www.nccn.org. 10.6004/jnccn.2204.0023 38754467

[14] Zhou C , Tang KJ, Cho BC, Liu B, Paz-Ares L, Cheng S, . Amivantamab plus chemotherapy in NSCLC with EGFR exon 20 insertions. N Engl J Med 2023;389:2039–51. 10.1056/NEJMoa2306441 37870976

[15] U.S. Food and Drug Administration . FDA grants accelerated approval to sunvozertinib for metastatic non-small cell lung cancer with EGFR exon 20 insertion mutations. Silver Spring (MD): U.S. Food and Drug Administration; 2025 [cited 2025 Jul 14]. Available from: https://www.fda.gov/drugs/resources-information-approved-drugs/fda-grants-accelerated-approval-sunvozertinib-metastatic-non-small-cell-lung-cancer-egfr-exon-20.

[16] Wells L , Qin A. Treatment of non-small cell lung cancer with atypical EGFR mutations. Curr Treat Options Oncol 2023;24:1802–14. 10.1007/s11864-023-01159-z 38095779

[17] Cheunkarndee T , Guo MZ, Houseknecht S, Feliciano JL, Hann CL, Lam VK, . First-line osimertinib for lung cancer with uncommon EGFR exon 19 mutations and EGFR compound mutations. JTO Clin Res Rep 2024;5:100686. 10.1016/j.jtocrr.2024.100686 PMC11225339 38975613

[18] Nguyen D , Shum E, Baik CS, Patel JD, Jänne P, Patel JD, . Emerging phase 1 data of BLU-451 in advanced NSCLC with EGFR exon 20 insertions. In: 2023 American Society of Clinical Oncology (ASCO) Annual Meeting; 2023 Jun 2–6; Chicago (IL), and online. Poster 52.

[19] Spira A , Yu H, Sun L, Nguyen D, Pearson P, Shim-Lopez J, . A phase 1/2 study of BLU-451, a central nervous system (CNS) penetrant, small molecule inhibitor of EGFR, in incurable advanced cancers with EGFR exon 20 insertion (ex20ins) mutations. In: 2022 American Society of Clinical Oncology (ASCO) Annual Meeting; 2022 Jun 3–7; Chicago (IL), and online. Abstract TPS9155.

[20] Gruff E , Kuang S, Pandey A, Shah H, Xie T, inventor; Blueprint Medicines Corp, assignee. Salt and crystal forms of an epidermal growth factor receptor inhibitor. United States patent US 18/862,859. 2023 [cited 2025 Dec 17]. Available from: https://patentscope.wipo.int/search/en/detail.jsf?docId=WO2023215431&_cid=P11-M4MQ3X-60998-1.WO/2023/215431(PCT/US2023/020925).

[21] Anastassiadis T , Deacon SW, Devarajan K, Ma H, Peterson JR. Comprehensive assay of kinase catalytic activity reveals features of kinase inhibitor selectivity. Nat Biotechnol 2011;29:1039–45. 10.1038/nbt.2017 PMC3230241 22037377

[22] Eno MS , Brubaker JD, Campbell JE, De Savi C, Guzi TJ, Williams BD, . Discovery of BLU-945, a reversible, potent, and wild-type-sparing next-generation EGFR mutant inhibitor for treatment-resistant non-small-cell lung cancer. J Med Chem 2022;65:9662–77. 10.1021/acs.jmedchem.2c00704 PMC9340769 35838760

[23] Ballard P , Yates JW, Yang Z, Kim DW, Yang JC, Cantarini M, . Preclinical comparison of osimertinib with other EGFR-TKIs in EGFR-mutant NSCLC brain metastases models, and early evidence of clinical brain metastases activity. Clin Cancer Res 2016;22:5130–40. 10.1158/1078-0432.CCR-16-0399 27435396

[24] Colclough N , Chen K, Johnström P, Strittmatter N, Yan Y, Wrigley GL, . Preclinical comparison of the blood-brain barrier permeability of osimertinib with other EGFR TKIs. Clin Cancer Res 2021;27:189–201. 10.1158/1078-0432.CCR-19-1871 33028591

[25] Cross DA , Ashton SE, Ghiorghiu S, Eberlein C, Nebhan CA, Spitzler PJ, . AZD9291, an irreversible EGFR TKI, overcomes T790M-mediated resistance to EGFR inhibitors in lung cancer. Cancer Discov 2014;4:1046–61. 10.1158/2159-8290.CD-14-0337 PMC4315625 24893891

[26] Johnson & Johnson . RYBREVANT - use in central nervous system (CNS) metastases. 2025 [cited 2025 Dec 17]. Available from: https://www.jnjmedicalconnect.com/products/rybrevant/medical-content/rybrevant-use-in-central-nervous-system-cns-metastases#bibRef0e87887de49424ac4bd3e0ee427ae7630.

[27] ZEGFROVY^TM^ (sunvozertinib) . Prescribing information. Shanghai (China): Dizal Pharmaceuticals; 2025.

[28] Lim CK , Wei YF, Tsai MS, Chen KY, Shih JY, Yu CJ. Treatment effectiveness and tolerability of afatinib at different doses in patients with EGFR-mutated lung adenocarcinoma: how low can we go? Eur J Cancer 2018;103:32–40. 10.1016/j.ejca.2018.07.128 30199768

[29] National Comprehensive Cancer Network . Metastatic non-small cell lung cancer. Plymouth Meeting (PA): National Comprehensive Cancer Network; 2025 [cited 2025 Nov 25]. Available from: https://www.nccn.org.

[30] Pfeil AJ , Hale JD, Zhang TS, Wakayama K, Miyazaki I, Odintsov I, . Preclinical evaluation of targeted therapies for central nervous system metastases. Dis Model Mech 2024;17:dmm050836. 10.1242/dmm.050836 PMC11463968 39344915

[31] Zou L , Chien HC, Pade D, Li Y, Nguyen M, Bhamidipati RK, . Considerations in K_p,uu,brain_-based strategy for selecting CNS-targeted drug candidates with sufficient target coverage and substantial pharmacodynamic effect. AAPS J 2025;27:52. 10.1208/s12248-025-01035-8 40021548

[32] Bernstein E , Luo J, Wang K, Negrao MV, Jänne PA, Sabari JK. Safety and intracranial activity of adagrasib in patients with KRAS^G12C^-mutated non-small-cell lung cancer and untreated CNS metastases in the KRYSTAL-1 Trial: a case series. JCO Precis Oncol 2024;8:e2300447. 10.1200/PO.23.00447 PMC10860949 38330263

[33] Negrao MV , Spira AI, Heist RS, Jänne PA, Pacheco JM, Weiss J, . Intracranial efficacy of adagrasib in patients from the KRYSTAL-1 trial with KRAS^G12C^-mutated non-small-cell lung cancer who have untreated CNS metastases. J Clin Oncol 2023;41:4472–7. 10.1200/JCO.23.00046 PMC10553074 37327468

[34] Sabari JK , Velcheti V, Shimizu K, Strickland MR, Heist RS, Singh M, . Activity of adagrasib (MRTX849) in brain metastases: preclinical models and clinical data from patients with KRASG12C-mutant non-small cell lung cancer. Clin Cancer Res 2022;28:3318–28. 10.1158/1078-0432.CCR-22-0383 PMC9662862 35404402

[35] Wang M , Yang JC, Mitchell PL, Fang J, Camidge DR, Nian W, . Sunvozertinib, a selective EGFR inhibitor for previously treated non-small cell lung cancer with EGFR exon 20 insertion mutations. Cancer Discov 2022;12:1676–89. 10.1158/2159-8290.CD-21-1615 PMC9262839 35404393

[36] Swaih AM , Talbot S, Savoca A, Thorpe H, Flemington V, Phillips B, . AZ14289671 is a highly selective and blood-brain barrier penetrant irreversible TKI that targets EGFRExon20 insertions. Cell Rep Med 2025;6:102305. 10.1016/j.xcrm.2025.102305 PMC12490212 40858103

[37] Wu J , Wu H, Zhou F, Wang Y, Wang C, Ji J. Blood-brain barrier alterations as an initiator of brain metastasis from lung cancer: a new take on an old story. Cancer Gene Ther 2025;32:809–16. 10.1038/s41417-025-00916-6 40456826

[38] Zheng MM , Xia Y, Pan K, Sun F, Tan AC, Dong XR, . The evolving landscape of leptomeningeal metastases from NSCLC: an international, contemporary, multicenter cohort study. Ann Oncol 2026;37:104–16. 10.1016/j.annonc.2025.09.140 PMC12981536 41125209

[39] Amivantamab OK’d for EGFR-mutant NSCLC. Cancer Discov 2021;11:1604. 10.1158/2159-8290.CD-NB2021-0351 34083225

[40] Vijayaraghavan S , Lipfert L, Chevalier K, Bushey BS, Henley B, Lenhart R, . Amivantamab (JNJ-61186372), an Fc enhanced EGFR/cMet bispecific antibody, induces receptor downmodulation and antitumor activity by monocyte/macrophage trogocytosis. Mol Cancer Ther 2020;19:2044–56. 10.1158/1535-7163.MCT-20-0071 32747419

[41] Yun J , Lee SH, Kim SY, Jeong SY, Kim JH, Pyo KH, . Antitumor activity of amivantamab (JNJ-61186372), an EGFR-MET bispecific antibody, in diverse models of EGFR exon 20 insertion-driven NSCLC. Cancer Discov 2020;10:1194–209. 10.1158/2159-8290.CD-20-0116 32414908

