# Supplementary material for: BLU-451, a CNS-Active, Potent, and Selective Small-Molecule Inhibitor against Uncommon EGFR Mutations
Source: Mol Cancer Ther. 2026 May 13;25(11):1828–42. doi: 10.1158/1535-7163.MCT-25-1056 (PMC13728808; doi:10.1158/1535-7163.MCT-25-1056)
Supplement: Supplementary file 1 [file mct-25-1056_figure_s10_supps10.pdf]

Supplementary Figure S10.

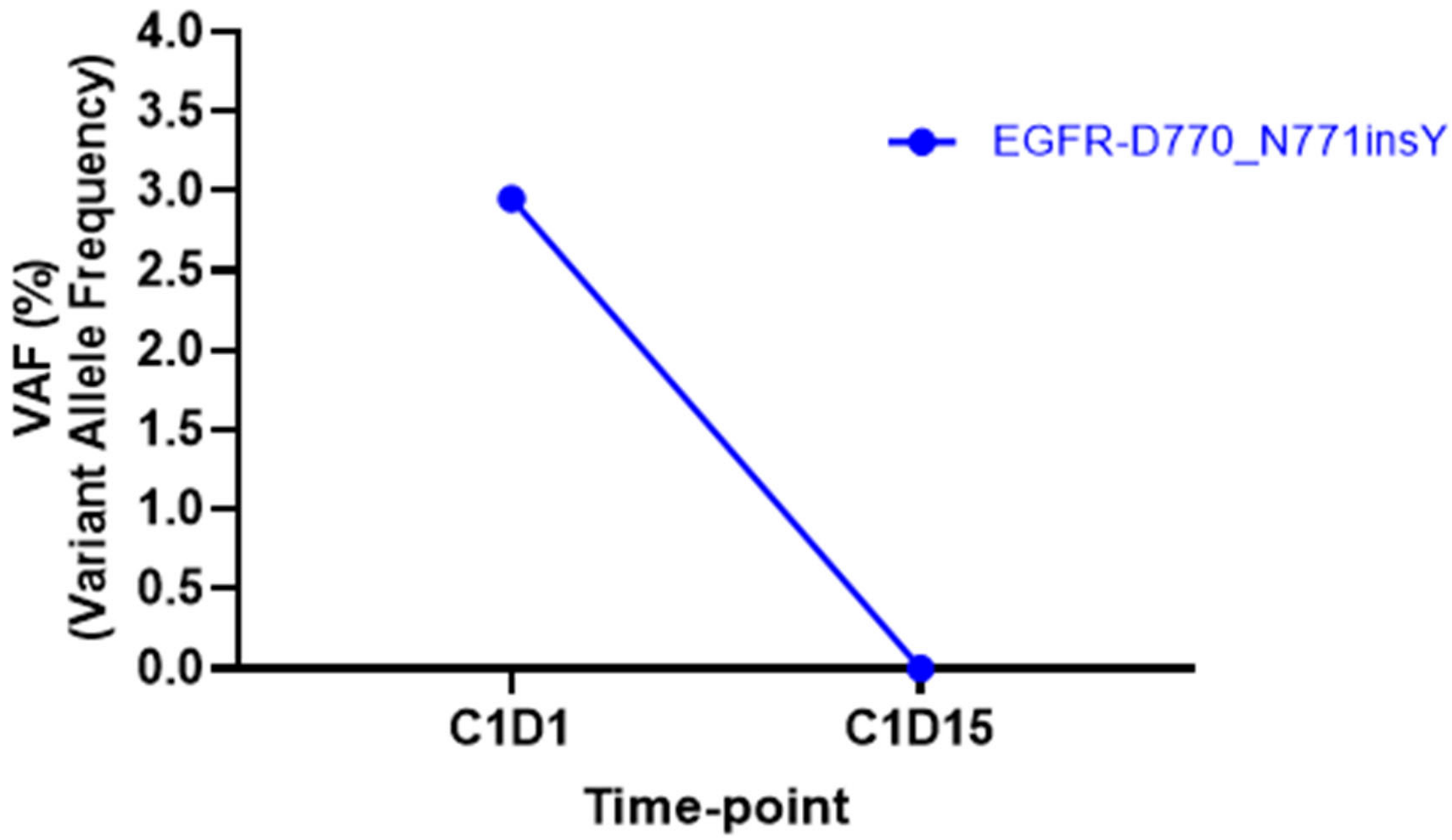

Supplementary Figure S10. ctDNA profiling in a patient with stage IVB NSCLC with *EGFR* exon 20 insertion mutation. *EGFR* D770_N771insY ctDNA at C1D15 compared with C1D1 of treatment with BLU-451 at 200 mg BID. At C1D15, the VAF fell below the limit of detection of the assay. C1D1, cycle 1, day 1; C1D15, cycle 1, day 15. BID, twice daily; ctDNA, circulating tumor DNA; EGFR, epidermal growth factor receptor; NSCLC, non-small cell lung cancer; VAF, variant allele frequency.
