# Supplementary material for: BLU-451, a CNS-Active, Potent, and Selective Small-Molecule Inhibitor against Uncommon EGFR Mutations
Source: Mol Cancer Ther. 2026 May 13;25(11):1828–42. doi: 10.1158/1535-7163.MCT-25-1056 (PMC13728808; doi:10.1158/1535-7163.MCT-25-1056)
Supplement: Supplementary file 2 [file mct-25-1056_figure_s1_supps1.pdf]

Supplementary Figure S1.

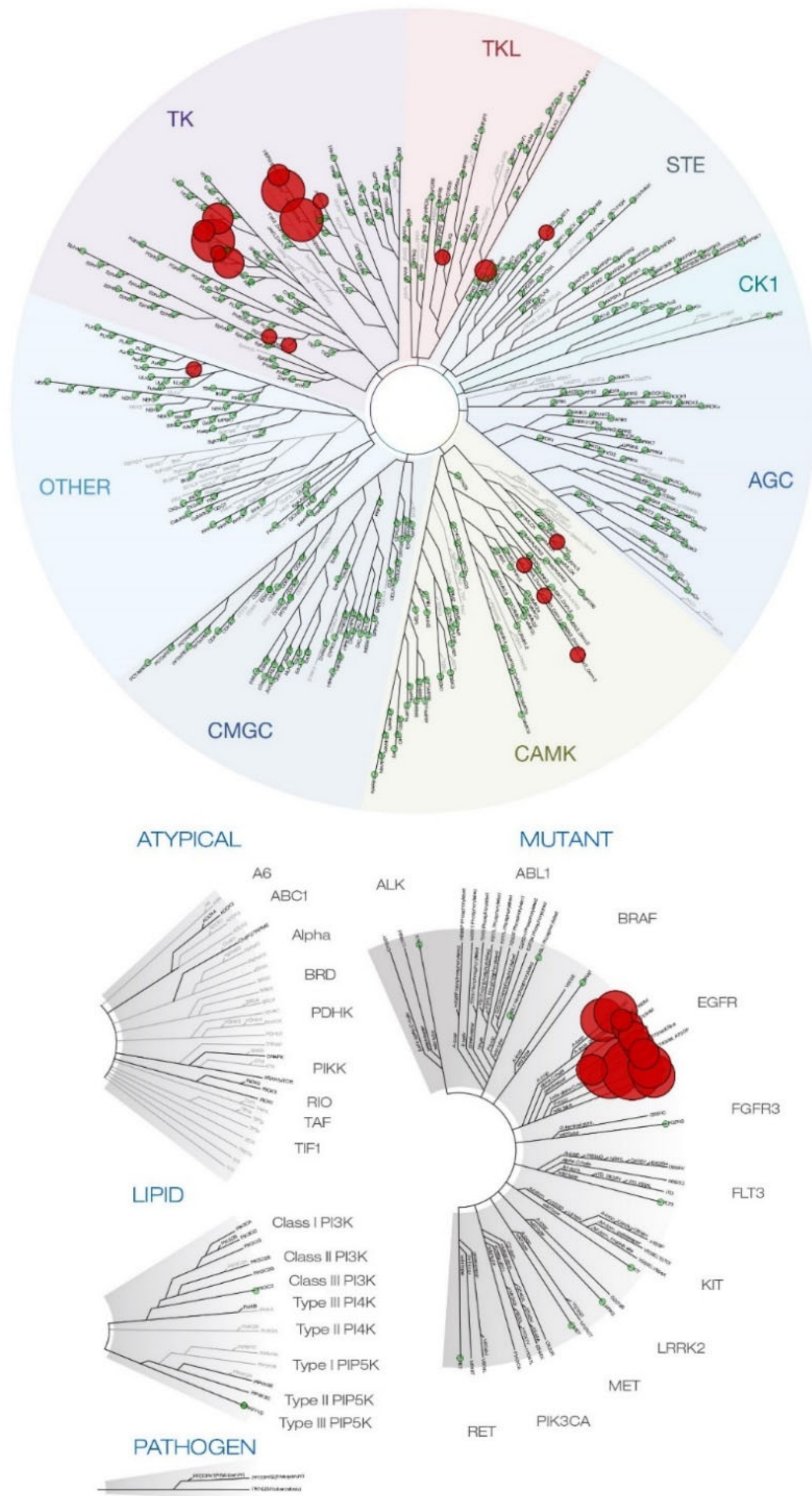

Supplementary Figure S1. Kinome tree showing the kinase selectivity of BLU-451 as determined by the KINOMEscan binding assay for BLU-451 at 1 µM. Image generated using TREE*spot*™ software tool.
