# Supplementary material for: BLU-451, a CNS-Active, Potent, and Selective Small-Molecule Inhibitor against Uncommon EGFR Mutations
Source: Mol Cancer Ther. 2026 May 13;25(11):1828–42. doi: 10.1158/1535-7163.MCT-25-1056 (PMC13728808; doi:10.1158/1535-7163.MCT-25-1056)
Supplement: Supplementary file 3 [file mct-25-1056_figure_s2_supps2.pdf]

**Supplementary Figure S2.**

**A**

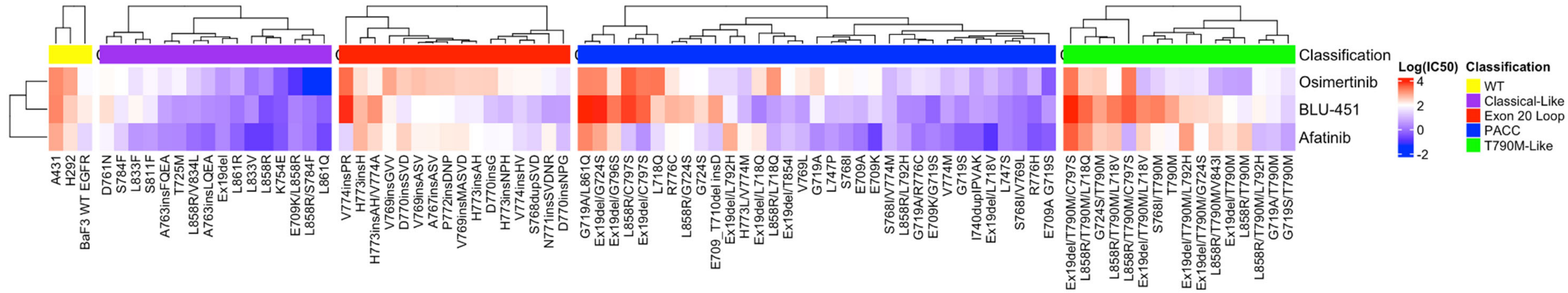


**B**

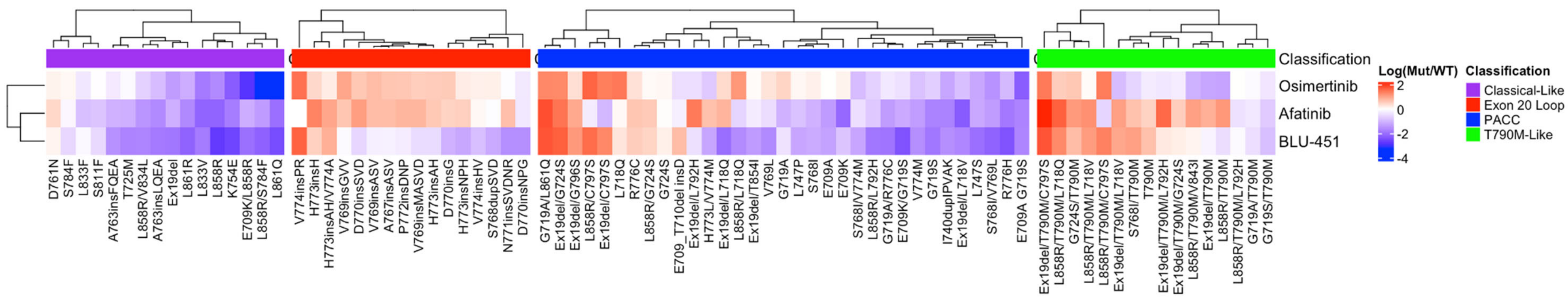


**Supplementary Figure S2.** BLU-451 potency and selectivity against Ba/F3 cells expressing EGFR variants from different subtypes. Heatmap with unsupervised hierarchical clustering of the $log_{10}$-transformed $IC_{50}$ values (A) and $IC_{50}$ ratio between mutant and WT EGFR (mut/WT) (B) for Ba/F3 cells expressing EGFR variants from the classical-like, exon 20 loop insertion, PACC, and T790M-like subtype (2) treated with BLU-451, osimertinib, or afatinib. The antiproliferation $IC_{50}$ values for each cell line and treatment were determined as described in the Methods section. The mut/WT ratio was calculated by dividing $IC_{50}$ values by the $IC_{50}$ obtained with the Ba/F3 cell line expressing WT EGFR. Data represent the median of *n* = 3 replicates. EGFR, epidermal growth factor receptor; $IC_{50}$, half maximal inhibitory concentration; PACC, P-loop and αC-helix compressing EGFR.
