# Supplementary material for: BLU-451, a CNS-Active, Potent, and Selective Small-Molecule Inhibitor against Uncommon EGFR Mutations
Source: Mol Cancer Ther. 2026 May 13;25(11):1828–42. doi: 10.1158/1535-7163.MCT-25-1056 (PMC13728808; doi:10.1158/1535-7163.MCT-25-1056)
Supplement: Supplementary file 4 [file mct-25-1056_figure_s3_supps3.pdf]

Supplementary Figure S3.

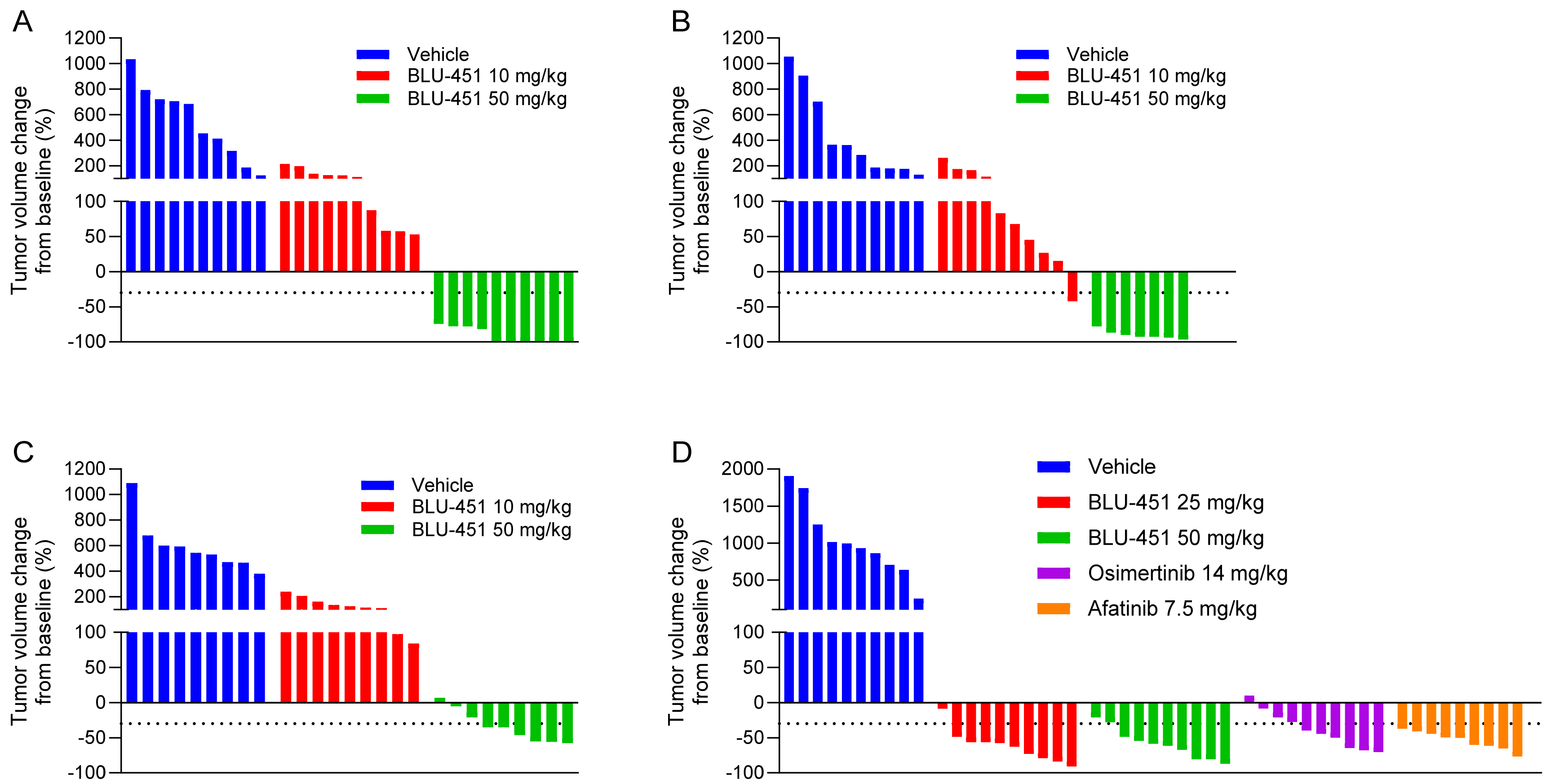

Supplementary Figure S3. Effect of BLU-451 treatment on tumor growth in uncommon EGFR PDX and Ba/F3 mouse tumor models. Percent change from baseline for tumor growth in **A,** the LU0387 PDX model carrying the *EGFR* exon 20 insertion H773insNPH; **B,** the LXFE2478 PDX model carrying the *EGFR* exon 20 insertion V769insASV; **C,** the *EGFR* exon 20 insertion V769insASV Ba/F3 tumor model; and **D,** the *EGFR* G719S Ba/F3 tumor model. Mice were orally dosed once daily with BLU-451 at 10, 25, or 50 mg/kg, osimertinib at 14 mg/kg, or afatinib at 7.5 mg/kg as indicated. Data represent individual animals. The threshold for tumor regression (defined as >30% reduction in tumor growth) is represented by the dotted lines. EGFR, epidermal growth factor receptor; PDX, patient-derived xenograft.
