# Supplementary material for: BLU-451, a CNS-Active, Potent, and Selective Small-Molecule Inhibitor against Uncommon EGFR Mutations
Source: Mol Cancer Ther. 2026 May 13;25(11):1828–42. doi: 10.1158/1535-7163.MCT-25-1056 (PMC13728808; doi:10.1158/1535-7163.MCT-25-1056)
Supplement: Supplementary file 6 [file mct-25-1056_figure_s5_supps5.pdf]

Supplementary Figure S5.

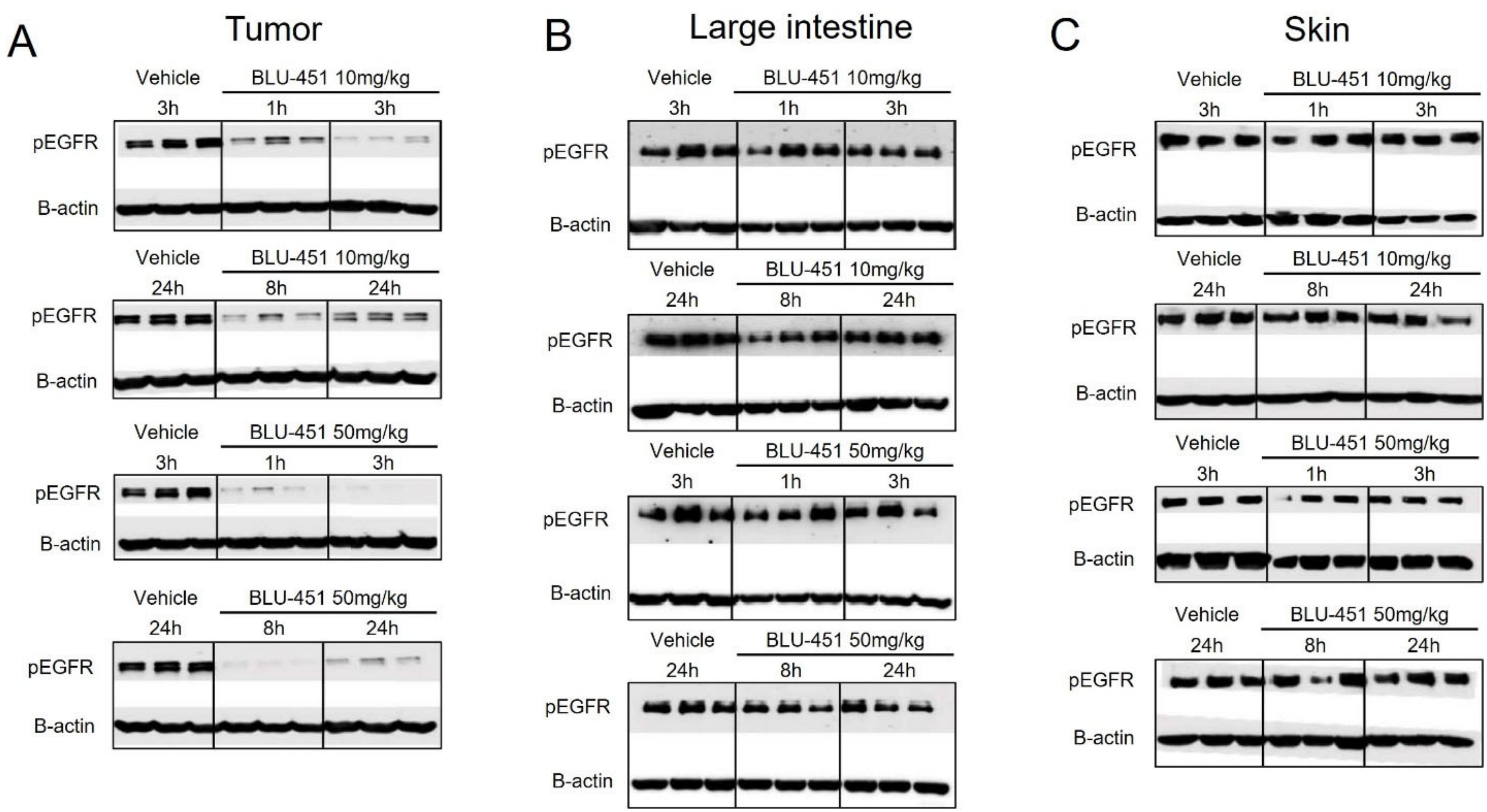

Supplementary Figure S5. Western blot analysis of EGFR phosphorylation in the A769insASV Ba/F3 mouse tumor model. See Methods section for details. Western blot for pEGFR and β-actin in **A,** tumor; **B,** large intestine; and **C,** skin tissues isolated from A769insASV Ba/F3 mice treated with vehicle or BLU-451 at 10 mg/kg or 50 mg/kg for 1, 3, 8, or 24 hours. EGFR, epidermal growth factor receptor; pEGFR, phosphorylated EGFR.
