# Supplementary material for: BLU-451, a CNS-Active, Potent, and Selective Small-Molecule Inhibitor against Uncommon EGFR Mutations
Source: Mol Cancer Ther. 2026 May 13;25(11):1828–42. doi: 10.1158/1535-7163.MCT-25-1056 (PMC13728808; doi:10.1158/1535-7163.MCT-25-1056)
Supplement: Supplementary file 7 [file mct-25-1056_figure_s6_supps6.pdf]

Supplementary Figure S6.

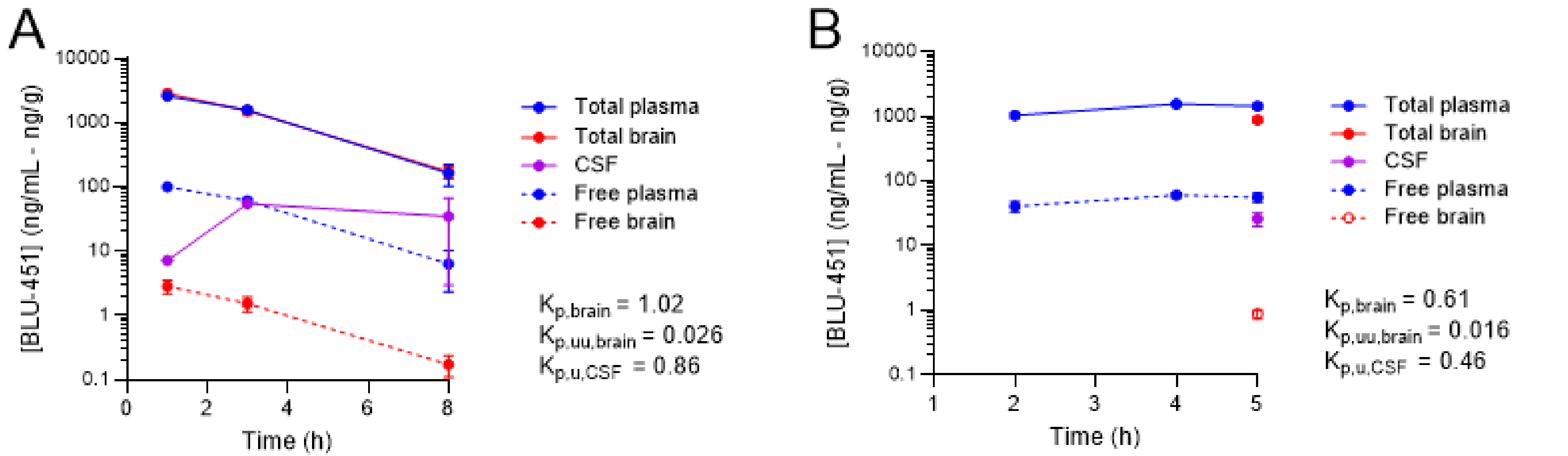

Supplementary Figure S6. Pharmacokinetics of plasma BLU-451 after an oral dose of BLU-451 at 30 mg/kg (A) or intravenous continuous infusion of BLU-451 at 20 mg/kg (B) in 7- to 8-week-old male rats. The $K_{p,brain}$, $K_{p,uu,brain}$, and $K_{p,u,CSF}$ values were determined as described in the Methods section. Data represent the average of 3 animals ± SEM.
