# Supplementary material for: BLU-451, a CNS-Active, Potent, and Selective Small-Molecule Inhibitor against Uncommon EGFR Mutations
Source: Mol Cancer Ther. 2026 May 13;25(11):1828–42. doi: 10.1158/1535-7163.MCT-25-1056 (PMC13728808; doi:10.1158/1535-7163.MCT-25-1056)
Supplement: Supplementary file 8 [file mct-25-1056_figure_s7_supps7.pdf]

Supplementary Figure S7.

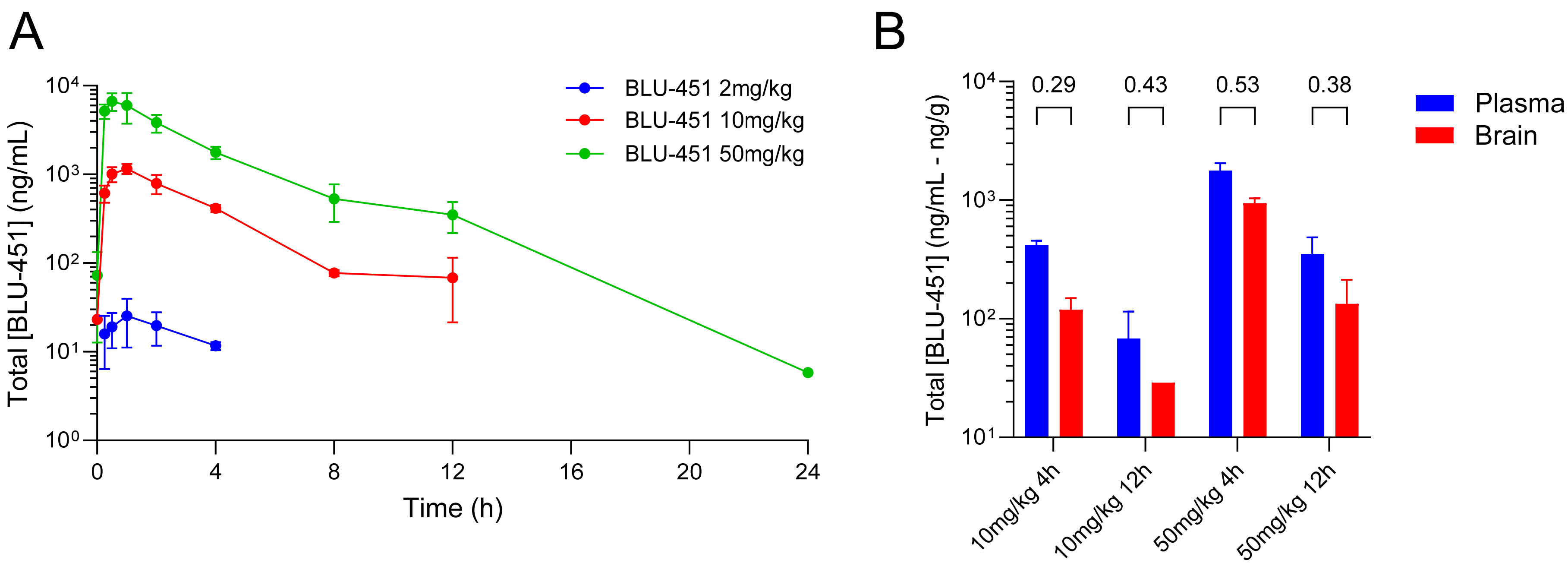

Supplementary Figure S7. BLU-451 pharmacokinetic analysis in BALB/c mice after repeated dosing. **A,** Pharmacokinetic of plasma BLU-451 after an oral dose of BLU-451 at 2, 10, or 50 mg/kg in 7- to 8-week-old male BALB/c mice following 5 days of oral once-daily dosing. **B,** BLU-451 plasma and brain levels at 4 and 12 hours. Data represent the average of 3 animals ± SEM. The brain-to-plasma ratio for each time point is indicated above the bars in **B**.
