# Supplementary material for: BLU-451, a CNS-Active, Potent, and Selective Small-Molecule Inhibitor against Uncommon EGFR Mutations
Source: Mol Cancer Ther. 2026 May 13;25(11):1828–42. doi: 10.1158/1535-7163.MCT-25-1056 (PMC13728808; doi:10.1158/1535-7163.MCT-25-1056)
Supplement: Supplementary file 9 [file mct-25-1056_figure_s8_supps8.pdf]

Supplementary Figure S8A.

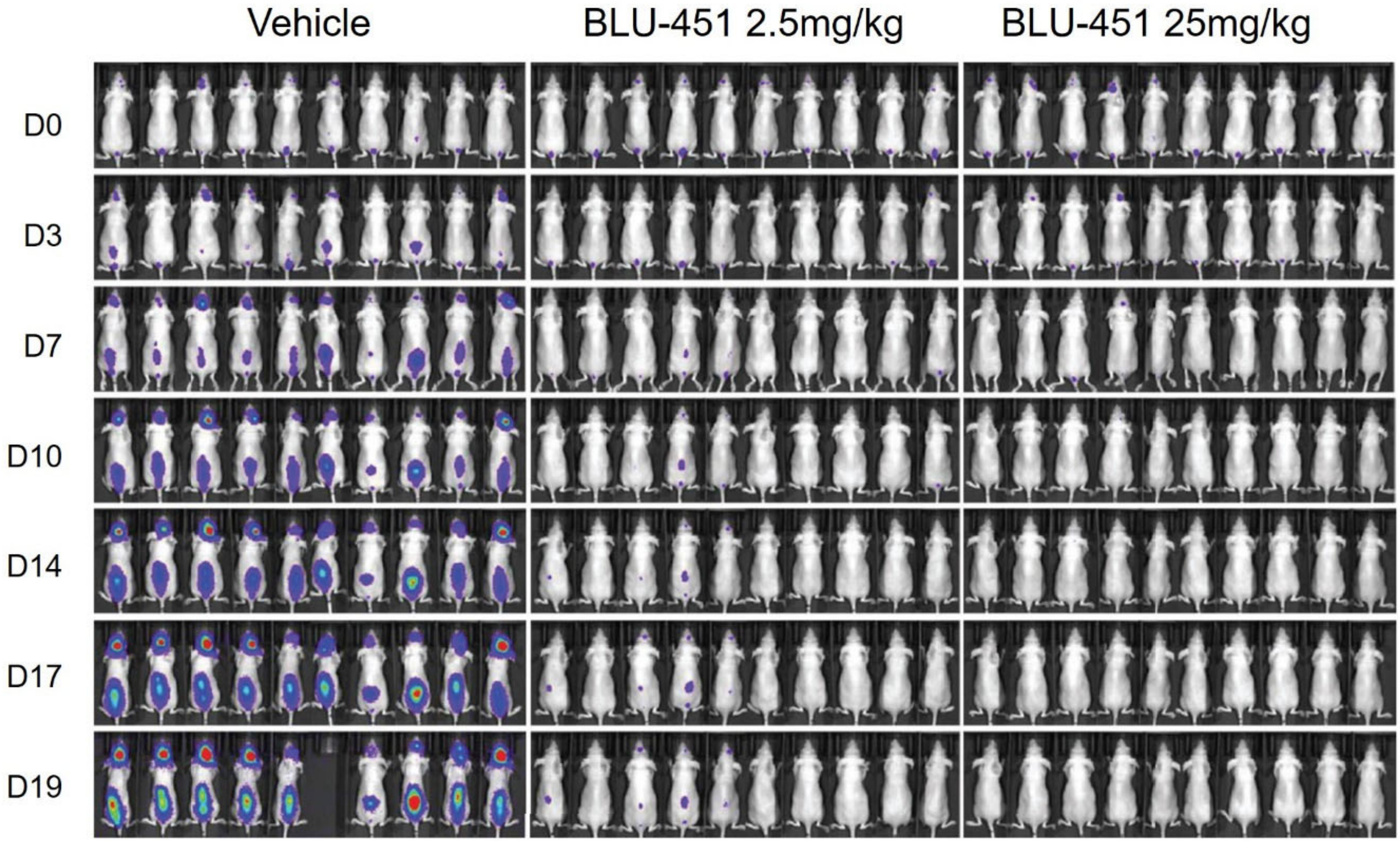

Supplementary Figure S8B.

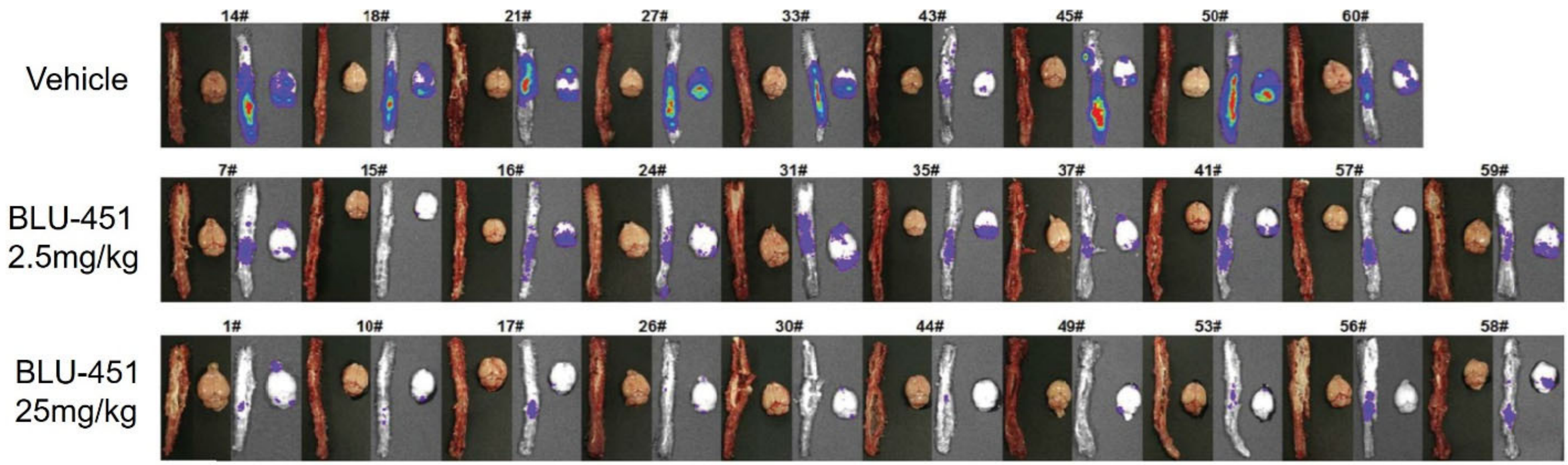

Supplementary Figure S8. Images of the PC9-luciferase intracranial model. **A,** Whole-body images. **B,** *Ex vivo* images of spinal cord (left) and brain (right) samples.
