# Supplementary material for: BLU-451, a CNS-Active, Potent, and Selective Small-Molecule Inhibitor against Uncommon EGFR Mutations
Source: Mol Cancer Ther. 2026 May 13;25(11):1828–42. doi: 10.1158/1535-7163.MCT-25-1056 (PMC13728808; doi:10.1158/1535-7163.MCT-25-1056)
Supplement: Supplementary file 11 [file mct-25-1056_supplementary_tables_suppst.docx]

# Supplementary Tables

**Supplementary Table S1. Kinase profiling binding assay with 1 µM BLU-451**

| **KINOMEscan gene symbol** | **Percent of control** | **KINOMEscan gene symbol** | **Percent of control** |
| --- | --- | --- | --- |
| EGFR(E746-A750del) | 0 | CLK2 | 59 |
| EGFR(G719S) | 0 | FLT3 | 62 |
| EGFR(L861Q) | 0.35 | TSSK1B | 64 |
| TEC | 0.4 | SIK2 | 66 |
| EGFR | 0.55 | LTK | 67 |
| EGFR(L858R) | 0.6 | MARK3 | 69 |
| ERBB4 | 0.65 | ABL1(Q252H)-nonphosphorylated | 70 |
| EGFR(G719C) | 0.9 | GAK | 70 |
| BTK | 1.2 | MAPKAPK2 | 72 |
| EGFR(L747-E749del, A750P) | 1.6 | ABL1(Y253F)-phosphorylated | 74 |
| BLK | 1.7 | AKT2 | 74 |
| EGFR(L747-S752del, P753S) | 2.4 | SRC | 74 |
| EGFR(L747-T751del,Sins) | 2.9 | YANK3 | 74 |
| EGFR(S752-I759del) | 4.5 | GSK3A | 75 |
| EGFR(T790M) | 4.7 | NIM1 | 75 |
| TXK | 5 | PRKCH | 75 |
| EGFR(L858R,T790M) | 6.7 | CLK4 | 76 |
| PAK3 | 7.8 | PIP5K2C | 76 |
| ERBB2 | 7.9 | ADCK4 | 77 |
| JAK3(JH1domain-catalytic) | 18 | IRAK3 | 77 |
| RSK1(Kin.Dom.2-C-terminal) | 18 | PRKX | 77 |
| BMX | 20 | ABL1-nonphosphorylated | 78 |
| RSK4(Kin.Dom.2-C-terminal) | 20 | ARK5 | 78 |
| TLK1 | 20 | GCN2(Kin.Dom.2,S808G) | 78 |
| PDGFRA | 26 | PKN1 | 78 |
| RSK3(Kin.Dom.2-C-terminal) | 30 | RPS6KA5(Kin.Dom.1-N-terminal) | 78 |
| DAPK1 | 31 | SIK | 78 |
| MST3 | 31 | STK33 | 78 |
| PDGFRB | 32 | BRSK2 | 79 |
| ACVRL1 | 33 | CDKL1 | 79 |
| PFCDPK1(P.falciparum) | 43 | DYRK1B | 79 |
| RET(M918T) | 44 | PRKD3 | 79 |
| DDR1 | 45 | CDK2 | 80 |
| ABL1(H396P)-nonphosphorylated | 46 | KIT(A829P) | 80 |
| RET | 46 | MLK2 | 80 |
| PLK2 | 48 | MAP4K2 | 81 |
| ROS1 | 49 | MKK7 | 81 |
| MLK1 | 51 | MYO3B | 81 |
| GRK4 | 56 | ABL2 | 82 |
| AKT3 | 58 | DLK | 82 |
| PRKCQ | 82 | CLK1 | 90 |
| LKB1 | 83 | DYRK1A | 90 |
| CDK8 | 84 | FGFR1 | 90 |
| CSNK1D | 84 | FLT3(ITD,F691L) | 90 |
| FLT3(D835V) | 84 | FLT3(R834Q) | 90 |
| TAK1 | 84 | IGF1R | 90 |
| CDK4 | 85 | JAK1(JH1domain-catalytic) | 90 |
| CDKL5 | 85 | MEK2 | 90 |
| CSNK1G2 | 85 | PAK2 | 90 |
| FLT3(D835H) | 85 | PIK3CA(H1047Y) | 90 |
| FRK | 85 | ROCK1 | 90 |
| RSK3(Kin.Dom.1-N-terminal) | 85 | RPS6KA4(Kin.Dom.1-N-terminal) | 90 |
| SRMS | 85 | SgK110 | 90 |
| CAMK2A | 86 | VRK2 | 90 |
| HPK1 | 86 | CSNK1A1L | 91 |
| KIT(L576P) | 86 | CSNK1G1 | 91 |
| PIK3C2G | 86 | ERK8 | 91 |
| PLK4 | 86 | FGFR4 | 91 |
| PRKG1 | 86 | FYN | 91 |
| ABL1(E255K)-phosphorylated | 87 | ITK | 91 |
| CLK3 | 87 | MARK4 | 91 |
| CSNK1G3 | 87 | MAST1 | 91 |
| MLK3 | 87 | SYK | 91 |
| CAMK4 | 88 | WNK4 | 91 |
| ERK2 | 88 | CDK5 | 92 |
| HIPK4 | 88 | CDK9 | 92 |
| LATS2 | 88 | EPHA3 | 92 |
| LCK | 88 | EPHB1 | 92 |
| MET(M1250T) | 88 | KIT | 92 |
| NDR2 | 88 | MEK1 | 92 |
| PIK3C2B | 88 | MEK3 | 92 |
| PIP5K1A | 88 | NEK9 | 92 |
| ROCK2 | 88 | PIKFYVE | 92 |
| RPS6KA5(Kin.Dom.2-C-terminal) | 88 | ABL1(M351T)-phosphorylated | 93 |
| SRPK3 | 88 | BIKE | 93 |
| CAMK1B | 89 | EPHA8 | 93 |
| PFTAIRE2 | 89 | KIT(D816H) | 93 |
| PKAC-alpha | 89 | KIT(V559D) | 93 |
| PKAC-beta | 89 | MET | 93 |
| PRKD2 | 89 | MTOR | 93 |
| NEK2 | 93 | RET(V804M) | 95 |
| PIK3CA(M1043I) | 93 | RSK1(Kin.Dom.1-N-terminal) | 95 |
| RSK2(Kin.Dom.1-N-terminal) | 93 | SRPK2 | 95 |
| SGK2 | 93 | STK16 | 95 |
| TGFBR2 | 93 | TAOK2 | 95 |
| BMPR2 | 94 | TGFBR1 | 95 |
| BRK | 94 | TIE2 | 95 |
| BRSK1 | 94 | ULK2 | 95 |
| CSNK2A1 | 94 | AURKA | 96 |
| DMPK | 94 | CDC2L1 | 96 |
| JNK1 | 94 | ERK1 | 96 |
| KIT(V559D,V654A) | 94 | FLT3(D835Y) | 96 |
| MARK2 | 94 | FLT3(N841I) | 96 |
| MET(Y1235D) | 94 | GRK3 | 96 |
| NEK11 | 94 | LZK | 96 |
| PHKG1 | 94 | NDR1 | 96 |
| PRP4 | 94 | NEK7 | 96 |
| RIPK2 | 94 | PCTK2 | 96 |
| TNNI3K | 94 | PYK2 | 96 |
| TRKB | 94 | RIOK3 | 96 |
| WEE1 | 94 | TAOK1 | 96 |
| WNK1 | 94 | TAOK3 | 96 |
| ABL1-phosphorylated | 95 | AKT1 | 97 |
| AURKB | 95 | AURKC | 97 |
| BUB1 | 95 | AXL | 97 |
| CAMKK2 | 95 | CIT | 97 |
| CSNK1E | 95 | DAPK2 | 97 |
| EPHA5 | 95 | ERBB3 | 97 |
| FLT1 | 95 | FER | 97 |
| HUNK | 95 | FLT3(ITD) | 97 |
| JNK3 | 95 | HIPK1 | 97 |
| MARK1 | 95 | JAK1(JH2domain-pseudokinase) | 97 |
| NEK3 | 95 | KIT(V559D,T670I) | 97 |
| NEK6 | 95 | MAP3K15 | 97 |
| PCTK1 | 95 | NIK | 97 |
| PIK3CA(C420R) | 95 | PIK3CA(E545A) | 97 |
| PIK3CB | 95 | PRKCI | 97 |
| PIK3CD | 95 | PRKD1 | 97 |
| RAF1 | 95 | SLK | 97 |
| RET(V804L) | 95 | STK35 | 97 |
| TNK1 | 97 | ABL1(Q252H)-phosphorylated | 100 |
| TRKC | 97 | ABL1(T315I)-nonphosphorylated | 100 |
| TTK | 97 | ABL1(T315I)-phosphorylated | 100 |
| VPS34 | 97 | ACVR1 | 100 |
| WEE2 | 97 | ACVR1B | 100 |
| ANKK1 | 98 | ACVR2A | 100 |
| CSNK2A2 | 98 | ACVR2B | 100 |
| DCAMKL3 | 98 | ADCK3 | 100 |
| DYRK2 | 98 | ALK | 100 |
| FGFR3(G697C) | 98 | ALK(C1156Y) | 100 |
| FLT4 | 98 | ALK(L1196M) | 100 |
| GSK3B | 98 | AMPK-alpha1 | 100 |
| MLCK | 98 | AMPK-alpha2 | 100 |
| MST2 | 98 | ASK1 | 100 |
| MYLK2 | 98 | ASK2 | 100 |
| PIM2 | 98 | BMPR1A | 100 |
| PIP5K1C | 98 | BMPR1B | 100 |
| WNK3 | 98 | BRAF | 100 |
| AAK1 | 99 | BRAF(V600E) | 100 |
| ABL1(H396P)-phosphorylated | 99 | CAMK1 | 100 |
| CAMK1G | 99 | CAMK1D | 100 |
| CAMK2G | 99 | CAMK2B | 100 |
| CAMKK1 | 99 | CAMK2D | 100 |
| CDK3 | 99 | CASK | 100 |
| CDKL3 | 99 | CDC2L2 | 100 |
| CSK | 99 | CDC2L5 | 100 |
| ERK5 | 99 | CDK11 | 100 |
| GRK2 | 99 | CDK4-cyclinD1 | 100 |
| HIPK3 | 99 | CDK4-cyclinD3 | 100 |
| NLK | 99 | CDK7 | 100 |
| PAK7 | 99 | CDKL2 | 100 |
| PKMYT1 | 99 | CHEK1 | 100 |
| SNARK | 99 | CHEK2 | 100 |
| TESK1 | 99 | CSF1R | 100 |
| TIE1 | 99 | CSF1R-autoinhibited | 100 |
| TNIK | 99 | CSNK1A1 | 100 |
| ABL1(F317I)-nonphosphorylated | 100 | CTK | 100 |
| ABL1(F317I)-phosphorylated | 100 | DAPK3 | 100 |
| ABL1(F317L)-nonphosphorylated | 100 | DCAMKL1 | 100 |
| ABL1(F317L)-phosphorylated | 100 | DCAMKL2 | 100 |
| DDR2 | 100 | KIT(D816V) | 100 |
| DMPK2 | 100 | KIT-autoinhibited | 100 |
| DRAK1 | 100 | LATS1 | 100 |
| DRAK2 | 100 | LIMK1 | 100 |
| EIF2AK1 | 100 | LIMK2 | 100 |
| EPHA1 | 100 | LOK | 100 |
| EPHA2 | 100 | LRRK2 | 100 |
| EPHA4 | 100 | LRRK2(G2019S) | 100 |
| EPHA6 | 100 | LYN | 100 |
| EPHA7 | 100 | MAK | 100 |
| EPHB2 | 100 | MAP3K1 | 100 |
| EPHB3 | 100 | MAP3K2 | 100 |
| EPHB4 | 100 | MAP3K3 | 100 |
| EPHB6 | 100 | MAP3K4 | 100 |
| ERK3 | 100 | MAP4K3 | 100 |
| ERK4 | 100 | MAP4K4 | 100 |
| ERN1 | 100 | MAP4K5 | 100 |
| FAK | 100 | MAPKAPK5 | 100 |
| FES | 100 | MEK4 | 100 |
| FGFR2 | 100 | MEK5 | 100 |
| FGFR3 | 100 | MEK6 | 100 |
| FGR | 100 | MELK | 100 |
| FLT3(ITD,D835V) | 100 | MERTK | 100 |
| FLT3(K663Q) | 100 | MINK | 100 |
| FLT3-autoinhibited | 100 | MKNK1 | 100 |
| GRK1 | 100 | MKNK2 | 100 |
| GRK7 | 100 | MRCKA | 100 |
| HASPIN | 100 | MRCKB | 100 |
| HCK | 100 | MST1 | 100 |
| HIPK2 | 100 | MST1R | 100 |
| ICK | 100 | MST4 | 100 |
| IKK-alpha | 100 | MUSK | 100 |
| IKK-beta | 100 | MYLK | 100 |
| IKK-epsilon | 100 | MYLK4 | 100 |
| INSR | 100 | MYO3A | 100 |
| INSRR | 100 | NEK1 | 100 |
| IRAK1 | 100 | NEK10 | 100 |
| IRAK4 | 100 | NEK4 | 100 |
| JAK2(JH1domain-catalytic) | 100 | NEK5 | 100 |
| JNK2 | 100 | OSR1 | 100 |
| p38-alpha | 100 | S6K1 | 100 |
| p38-beta | 100 | SBK1 | 100 |
| p38-delta | 100 | SGK | 100 |
| p38-gamma | 100 | SGK3 | 100 |
| PAK1 | 100 | SNRK | 100 |
| PAK4 | 100 | SRPK1 | 100 |
| PAK6 | 100 | STK36 | 100 |
| PCTK3 | 100 | STK39 | 100 |
| PDPK1 | 100 | TBK1 | 100 |
| PFPK5(P.falciparum) | 100 | TLK2 | 100 |
| PFTK1 | 100 | TNK2 | 100 |
| PHKG2 | 100 | TRKA | 100 |
| PIK3CA | 100 | TRPM6 | 100 |
| PIK3CA(E542K) | 100 | TSSK3 | 100 |
| PIK3CA(E545K) | 100 | TYK2(JH1domain-catalytic) | 100 |
| PIK3CA(H1047L) | 100 | TYK2(JH2domain-pseudokinase) | 100 |
| PIK3CA(I800L) | 100 | TYRO3 | 100 |
| PIK3CA(Q546K) | 100 | ULK1 | 100 |
| PIK3CG | 100 | ULK3 | 100 |
| PIK4CB | 100 | VEGFR2 | 100 |
| PIM1 | 100 | WNK2 | 100 |
| PIM3 | 100 | YANK1 | 100 |
| PIP5K2B | 100 | YANK2 | 100 |
| PKN2 | 100 | YES | 100 |
| PKNB(M.tuberculosis) | 100 | YSK1 | 100 |
| PLK1 | 100 | YSK4 | 100 |
| PLK3 | 100 | ZAK | 100 |
| PRKCD | 100 | ZAP70 | 100 |
| PRKCE | 100 |  |  |
| PRKG2 | 100 |  |  |
| PRKR | 100 |  |  |
| QSK | 100 |  |  |
| RIOK1 | 100 |  |  |
| RIOK2 | 100 |  |  |
| RIPK1 | 100 |  |  |
| RIPK4 | 100 |  |  |
| RIPK5 | 100 |  |  |
| RPS6KA4(Kin.Dom.2-C-terminal) | 100 |  |  |
| RSK2(Kin.Dom.2-C-terminal) | 100 |  |  |
| RSK4(Kin.Dom.1-N-terminal) | 100 |  |  |

**Supplementary Table S2. Kinase profiling enzymatic assay with 1 µM BLU-451**

| **Kinase** | **Percent of control** | **Kinase** | **Percent of control** |
| --- | --- | --- | --- |
| EGFR | 0.415 | LYN | 85.16 |
| BLK | 0.55 | NLK | 85.255 |
| TXK | 0.98 | MKK7 | 85.475 |
| JAK3 | 1.25 | CAMKK1 | 85.835 |
| ITK | 1.43 | SIK2 | 85.9 |
| ERBB4/HER4 | 1.49 | PKCg | 86.26 |
| BTK | 2.515 | SNRK | 86.495 |
| BMX/ETK | 3.005 | EPHA5 | 87.055 |
| ERBB2/HER2 | 6.59 | ULK1 | 87.065 |
| TEC | 11.865 | VRK1 | 87.415 |
| PLK2 | 17.82 | TBK1 | 87.515 |
| RET | 20.715 | PBK/TOPK | 87.565 |
| DDR1 | 38.345 | CDK3/cyclin E | 87.67 |
| YES/YES1 | 42.615 | PKN1/PRK1 | 87.825 |
| FGR | 56.92 | FGFR2 | 87.91 |
| ABL1 | 59.065 | BMPR2 | 87.975 |
| ACK1 | 63.195 | STK25/YSK1 | 88.18 |
| RIPK4 | 65.47 | TYK2 | 88.185 |
| LCK | 66.14 | PAK4 | 88.25 |
| c-Src | 67.73 | MSSK1/STK23 | 88.38 |
| SIK1 | 69.16 | BRK | 88.48 |
| PDGFRa | 69.86 | TNK1 | 88.9 |
| MLK1/MAP3K9 | 71.09 | DDR2 | 88.98 |
| ABL2/ARG | 73.325 | STK33 | 89.215 |
| FYN | 73.42 | FGFR3 | 89.295 |
| MST3/STK24 | 75.38 | CSK | 89.57 |
| CK1epsilon | 76.135 | FMS | 89.775 |
| EPHA6 | 78.635 | PKCmu/PRKD1 | 89.795 |
| HGK/MAP4K4 | 81.675 | EPHA4 | 90.095 |
| CDK2/cyclin A1 | 82.205 | IRR/INSRR | 90.185 |
| SGK3/SGKL | 82.3 | MARK4 | 90.49 |
| PDGFRb | 82.495 | CDK16/cyclin Y (PCTAIRE) | 90.725 |
| TNIK | 82.57 | PKG1b | 90.77 |
| TRKC | 82.985 | TESK1 | 91.07 |
| BRSK2 | 83.2 | MUSK | 91.145 |
| c-Kit | 83.925 | CDK5/p35 | 91.31 |
| TLK1 | 84.47 | PLK1 | 91.39 |
| MST1/STK4 | 84.915 | MST4 | 91.5 |
| MELK | 85.05 | GRK1 | 91.555 |
| IRAK4 | 91.655 | MSK1/RPS6KA5 | 94.57 |
| LIMK1 | 91.735 | DYRK1/DYRK1A | 94.645 |
| CK1G3 | 91.76 | TGFBR2 | 94.665 |
| P38d/MAPK13 | 91.775 | PAK2 | 94.665 |
| MLK3/MAP3K11 | 91.81 | FGFR1 | 94.75 |
| STK32B/YANK2 | 91.815 | MYO3b | 94.77 |
| CDK6/cyclin D1 | 91.855 | HPK1/MAP4K1 | 94.81 |
| WNK2 | 91.925 | GLK/MAP4K3 | 94.87 |
| STK16 | 92.265 | CDK5/P25 | 94.91 |
| CHK2 | 92.535 | PHKg2 | 95.08 |
| GSK3b | 92.615 | RSK3 | 95.08 |
| CK1g2 | 92.635 | KHS/MAP4K5 | 95.115 |
| TRKA | 92.715 | PKN2/PRK2 | 95.135 |
| EPHB2 | 92.935 | TESK2 | 95.225 |
| PKCb2 | 92.98 | MINK/MINK1 | 95.345 |
| CDK2/cyclin E2 | 93 | PKG1a | 95.35 |
| CDK8/cyclin C | 93.06 | ARAF | 95.38 |
| MARK3 | 93.195 | MAK | 95.435 |
| ASK1/MAP3K5 | 93.275 | PKCa | 95.49 |
| FLT3 | 93.325 | YSK4/MAP3K19 | 95.615 |
| CDK3/cyclin E2 | 93.36 | CHK1 | 95.75 |
| TTBK2 | 93.515 | RSK2 | 95.79 |
| p70S6K/RPS6KB1 | 93.525 | HIPK1 | 96.015 |
| CK1d | 93.57 | ERK7/MAPK15 | 96.11 |
| MNK2 | 93.635 | MARK1 | 96.125 |
| MASTL | 93.66 | AXL | 96.24 |
| DYRK1B | 93.715 | CAMK2b | 96.35 |
| c-MER | 93.745 | CDK7/cyclin H | 96.47 |
| STK22D/TSSK1 | 93.745 | PKCeta | 96.6 |
| SGK2 | 93.755 | ALK6/BMPR1B | 96.61 |
| COT1/MAP3K8 | 93.91 | PAK6 | 96.81 |
| Aurora B | 93.915 | PAK3 | 96.845 |
| CAMK2g | 93.915 | SBK1 | 96.895 |
| KSR2 | 93.965 | HIPK2 | 96.905 |
| CDK1/cyclin E | 93.98 | ZAK/MLTK | 96.955 |
| MEK1 | 94.25 | PKCb1 | 96.96 |
| TAOK1 | 94.305 | SSTK/TSSK6 | 96.96 |
| RSK1 | 94.48 | CDK1/cyclin A | 97.055 |
| CAMK4 | 94.545 | FGFR4 | 97.085 |
| DCAMKL1 | 97.09 | DAPK1 | 99.105 |
| EPHB3 | 97.09 | LRRK2 | 99.125 |
| BRSK1 | 97.13 | Haspin | 99.235 |
| ERK1 | 97.15 | FRK/PTK5 | 99.285 |
| JAK1 | 97.305 | STK21/CIT | 99.315 |
| ROCK2 | 97.31 | TSSK3/STK22C | 99.355 |
| GRK2 | 97.395 | CDK17/cyclin Y (PCTK2) | 99.365 |
| ALK2/ACVR1 | 97.405 | ALK4/ACVR1B | 99.4 |
| FLT1/VEGFR1 | 97.53 | PKA | 99.41 |
| DYRK2 | 97.535 | NEK5 | 99.445 |
| ROCK1 | 97.65 | PKCd | 99.525 |
| MYO3A | 97.725 | STK38L/NDR2 | 99.525 |
| CAMKK2 | 97.775 | EPHA7 | 99.53 |
| EPHB1 | 97.86 | CDK2/CYCLIN E | 99.545 |
| DCAMKL2 | 97.875 | EPHB4 | 99.57 |
| DMPK2 | 97.93 | DYRK3 | 99.64 |
| SLK/STK2 | 97.935 | LATS2 | 99.65 |
| PHKg1 | 97.96 | PKCzeta | 99.65 |
| ERK2/MAPK1 | 98.06 | EPHA2 | 99.74 |
| PAK5 | 98.13 | MARK2/PAR- | 99.745 |
| TYK1/LTK | 98.13 | HCK | 99.785 |
| FLT4/VEGFR3 | 98.155 | ERN2/IRE2 | 99.79 |
| NEK6 | 98.38 | GRK4 | 99.87 |
| SIK3 | 98.41 | SYK | 99.905 |
| GRK6 | 98.445 | PRKX | 99.965 |
| LOK/STK10 | 98.62 | GRK3 | 100.01 |
| CDK1/cyclin B | 98.625 | NEK9 | 100.095 |
| MST2/STK3 | 98.655 | CDK4/cyclin D1 | 100.105 |
| PKCtheta | 98.745 | NEK1 | 100.115 |
| LIMK2 | 98.755 | MKK4 | 100.13 |
| EPHA8 | 98.79 | ERK5/MAPK7 | 100.19 |
| DMPK | 98.795 | PIM1 | 100.215 |
| PKCepsilon | 98.795 | PIM2 | 100.22 |
| SRPK2 | 98.82 | CLK1 | 100.235 |
| RSK4 | 98.875 | AKT3 | 100.26 |
| TAOK2/TAO1 | 98.89 | GSK3a | 100.27 |
| TYRO3/SKY | 98.995 | MAPKAPK2 | 100.275 |
| CDK18/cyclin Y (PCTK3) | 99.045 | SRMS | 100.33 |
| EPHA3 | 99.045 | NEK4 | 100.45 |
| CLK3 | 100.54 | VRK2 | 102.675 |
| TSSK2 | 100.585 | CLK2 | 102.68 |
| IKKe/IKBKE | 100.605 | GRK7 | 102.77 |
| CDK2/cyclin A | 100.62 | TLK2 | 103.05 |
| PLK4/SAK | 100.63 | TRKB | 103.125 |
| ALK1/ACVRL1 | 100.665 | IGF1R | 103.26 |
| RAF1 | 100.76 | GRK5 | 103.28 |
| CAMK1g | 100.86 | ULK3 | 103.305 |
| CAMK2d | 100.87 | ULK2 | 103.315 |
| PDK1/PDPK1 | 100.89 | STK38/NDR1 | 103.33 |
| IR | 100.895 | MAPKAPK5/PRAK | 103.565 |
| TAOK3/JIK | 100.94 | GCK/MAP4K2 | 103.6 |
| AKT2 | 101.01 | NEK2 | 103.64 |
| WEE1 | 101.09 | FER | 103.77 |
| ROS/ROS1 | 101.1 | EPHA1 | 103.8 |
| JNK2 | 101.255 | MLCK/MYLK | 103.88 |
| P38b/MAPK11 | 101.265 | MRCKb/CDC42BPB | 103.89 |
| MEK3 | 101.29 | JNK1 | 103.955 |
| PKCnu/PRKD3 | 101.3 | CAMK1b | 103.965 |
| MEKK6 | 101.315 | PYK2 | 103.965 |
| ALK | 101.345 | LYN B | 104.025 |
| CDK4/cyclin D3 | 101.35 | KSR1 | 104.03 |
| LATS1 | 101.355 | PIM3 | 104.095 |
| CDK6/cyclin D3 | 101.41 | PKAcb | 104.155 |
| MEKK1 | 101.55 | CK2a | 104.315 |
| p70S6Kb/RPS6KB2 | 101.73 | MYLK4 | 104.325 |
| PKN3/PRK3 | 101.735 | MYLK3 | 104.335 |
| c-MET | 101.79 | HIPK4 | 104.49 |
| PKCIOTA | 101.82 | RIPK5 | 104.49 |
| AKT1 | 101.835 | JAK2 | 104.725 |
| PAK1 | 101.845 | MEKK2 | 104.77 |
| CDC7/DBF4 | 101.875 | LCK2/ICK | 104.86 |
| WNK3 | 101.945 | CLK4 | 104.875 |
| SNARK/NUAK2 | 102.135 | CK2a2 | 105.065 |
| WNK1 | 102.26 | CDK2/cyclin O | 105.255 |
| TAK1 | 102.28 | MEK2 | 105.44 |
| HIPK3 | 102.315 | STK39/STLK3 | 105.475 |
| JNK3 | 102.365 | SGK1 | 105.52 |
| SRPK1 | 102.61 | CK1a1 | 105.52 |
| KDR/VEGFR2 | 105.595 | MRCKa/CDC42BPA | 112.8 |
| PASK | 105.69 | PKD2/PRKD2 | 113.24 |
| MEK5 | 105.935 | CDK9/cyclin K | 113.375 |
| Aurora A | 106.145 | MEKK3 | 113.53 |
| OSR1/OXSR1 | 106.38 | IKKa/CHUK | 113.62 |
| NEK11 | 106.405 | CK1g1 | 113.675 |
| CAMK1d | 106.45 | PLK3 | 114.04 |
| FAK/PTK2 | 106.455 | DRAK1/STK17A | 114.445 |
| MLCK2/MYLK2 | 106.525 | MNK1 | 114.48 |
| BRAF | 106.575 | FES/FPS | 114.84 |
| MLK2/MAP3K10 | 106.58 | ALK3/BMPR1A | 115.195 |
| LKB1 | 107.16 | P38a/MAPK14 | 116.625 |
| RON/MST1R | 107.195 | MKK6 | 116.895 |
| IKKb/IKBKB | 107.2 | NIM1 | 117.01 |
| IRAK1 | 107.28 | TIE2/TEK | 117.22 |
| CDK9/cyclin T1 | 107.51 | CDK14/cyclin Y (PFTK1) | 118.64 |
| ALK5/TGFBR1 | 107.65 | DAPK2 | 118.805 |
| AURORA C | 108.295 | MAPKAPK3 | 119.49 |
| ARK5/NUAK1 | 108.5 | ERN1/IRE1 | 126.625 |
| NEK7 | 108.855 |  |  |
| MAST3 | 108.93 |  |  |
| NEK3 | 109.16 |  |  |
| P38g | 109.175 |  |  |
| CTK/MATK | 109.24 |  |  |
| CAMK1a | 109.26 |  |  |
| TTBK1 | 109.285 |  |  |
| MSK2/RPS6KA4 | 109.765 |  |  |
| RIPK2 | 110.385 |  |  |
| CDK9/cyclin T2 | 110.4 |  |  |
| PKAcg | 110.4 |  |  |
| ZAP70 | 110.565 |  |  |
| ZIPK/DAPK3 | 110.985 |  |  |
| DYRK4 | 111.15 |  |  |
| CK1a1L | 111.66 |  |  |
| CDK19/cyclin C | 112.205 |  |  |
| MLK4 | 112.24 |  |  |
| CAMK2a | 112.6 |  |  |
| STK32C/YANK3 | 112.6 |  |  |
| PKG2/PRKG2 | 112.725 |  |  |

**Supplementary Table S3**. Pharmacokinetic parameters of plasma BLU-451 following oral administration of BLU-451 at 2, 10, or 50 mg/kg in 7- to 8-week-old male BALB/c mice following 5 days of oral once-daily dosing.

| Dose | 2 mg/mL | 10 mg/mL | 50 mg/mL |
| --- | --- | --- | --- |
| t_1/2_ (h) | 2.68 | 3.06 | 2.34 |
| AUC_0-∞,u_ (ng.h/mL) | 2.63 | 103.2 | 547.9 |
| AUC_0-t,u_ (ng.h/mL) | 1.61 | 96.5 | 547.4 |
| C_max,u_ (ng/mL) | 0.57 | 26.2 | 150.5 |
| T_max_ (h) | 1.00 | 1.00 | 0.50 |

AUC_0-t,u_, area under the unbound plasma concentration-time curve from zero to last measurable time point; AUC_0-∞,u_, area under the unbound plasma concentration-time curve from time zero to infinity; t_1/2_, half-life; T_max_, time to the maximum observed plasma concentration; C_max,u_, maximum unbound concentration.

**Supplementary Table S4**. Pharmacokinetic parameters of plasma BLU-451 in Case 1 and Case 2 after 15 days of dosing

|  | Case 1 | Case 2 |
| --- | --- | --- |
| C_min,ss,u_ (ng/mL) | 2.85 | 2.00 |
| C_max,ss,u_ (ng/mL) | 21.7 | 6.54 |
| AUC_0-24,u_ (ng.h/mL) | 178.9 | 100.1 |
| T_max_ (h) | 2 | 1 |

AUC_0-24h,u_, area under the unbound plasma concentration-time curve from zero to 24h; T_max_, time to the maximum observed plasma concentration; C_max,ss,u_, maximum unbound concentration at steady-state; C_min,ss,u_, minimum unbound concentration at steady-state.
